# Risk factors for diabetic foot complications in type 2 diabetes—A systematic review

**DOI:** 10.1002/edm2.175

**Published:** 2020-08-17

**Authors:** Sophia Rossboth, Monika Lechleitner, Willi Oberaigner

**Affiliations:** ^1^ Medical Informatics and Technology Public Health, Health Services Research and Health Technology Assessment UMIT ‐ University for Health Sciences Hall in Troll Austria; ^2^ Department of Internal Medicine Hospital Hochzirl Zirl Austria

**Keywords:** diabetic foot, foot ulceration, lower extremity amputation, risk factors

## Abstract

**Aims:**

With increasing numbers of patients with type 2 diabetes mellitus (T2DM) worldwide, the number of associated diabetic foot complications might also increase. This systematic review was performed to summarize published data about risk factors for the diabetic foot (DF) syndrome in order to improve the identification of high‐risk patients.

**Materials and methods:**

Six electronic databases were searched for publications up to August 2019 using predefined stringent inclusion and exclusion criteria.

**Results:**

Of 9,476 identified articles, 31 articles from 28 different study populations fulfilled the criteria for our evaluation. The overall quality of the studies was good, and the risk of bias was low. There was large heterogeneity among the studies concerning study protocols and patient populations analysed. A total of 79 risk factors were analysed within this review. The majority of studies described a consistently positive association with different outcomes of interest related to DF for gender, peripheral neuropathy, retinopathy, nephropathy, poor glycaemic control, insulin use, duration of diabetes, smoking and height. For age, hypertension, dyslipidaemia and body mass index, the results remain inconsistent.

**Conclusion:**

A most up‐to‐date literature review resulted in glycaemic control and smoking as the only amenable risk factors with a consistently positive association for DF. Due to the high personal and financial burden associated with DF and the large heterogeneity among included studies, additional longitudinal studies in large patient populations are necessary to identify more modifiable risk factors that can be used in the prediction and prevention of DF complications.

## Graphical abstract

We performed a comprehensive and systematic literature search on six electronic databases using stringent inclusion and exclusion criteria to summarize published data on risk factors for the diabetic foot syndrome in type 2 diabetes patients. The findings of this review showed that gender, peripheral neuropathy, retinopathy, nephropathy, poor glycaemic control, insulin use, duration of diabetes, smoking and height are associated with diabetic foot complications, while for age, hypertension, dyslipidaemia and body mass index inconsistent results have been reported.

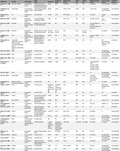

## 1 INTRODUCTION

Diabetes mellitus is one of the major health concerns of the 21st century. The number of patients with diabetes has been increasing steadily for the past three decades, and this increase will probably continue throughout the next decades: from an estimated 463 million patients between the age of 18 and 99 years affected in 2019 to an estimation of 700 million people in the same age group affected in 2045 worldwide. Diabetes accounts for approximately 4.2 million deaths annually and causes a tremendous financial burden on healthcare systems: in 2019, the global health care costs for diabetes totalled 760 billion US dollars for patients in the age group between 18 and 99 years.^1^, ^2^

Patients with diabetes face a high risk of developing serious adverse health conditions that shorten the life expectancy, lower the quality of life and increase medical care costs.^1^, ^3^ The diabetic foot (DF) syndrome is a serious diabetic late complication strongly related to diabetic neuropathy and peripheral artery disease. Tissue necrosis can result in a need for lower extremity amputation (LEA).^1^ According to the International Working Group on the Diabetic Foot (IWGDF), DF is defined as: ‘Infection, ulceration, or destruction of tissues of the foot of a person with currently or previously diagnosed diabetes mellitus, usually accompanied by neuropathy and/or peripheral arterial disease in the lower extremity’.^4^

Around 25% of all patients with diabetes develop foot complications during their course of disease.^5^ The condition constitutes a major cause for hospital admissions in people with diabetes, accounting for nearly 70% of all amputations conducted in the United States in 1997.^1^, ^6^, ^7^ Moreover, diabetic foot ulcers (FU) and amputations make up the most expensive diabetic late complication in terms of hospital costs.^8^ In the year after the first FU, the health expenditures for patients with diabetes with FUs are five times higher than for those without FUs and almost three times higher in the subsequent years. In 2007, one‐third of all costs for diabetes were linked solely to foot complications.^9^ Patients with diabetes suffering from FUs reveal a 10‐20 times higher risk for amputation than subjects without diabetes,^10^ and FUs are further associated with a higher mortality risk compared to those patients without foot complications.^11^ Approximately 1% of all patients with diabetes have to undergo lower limb amputation in high‐income countries, with the percentage being higher in low‐ and middle‐income countries.^1^ In addition, patients with a history of DF complications carry a higher risk of subsequent re‐ulcerations.^12^

DF conditions, especially with severe complications and the need for amputations, are one of the most serious and preventable diabetic late complications. Besides the efforts made on conducting regular foot examinations and the progress on risk classification systems, both prevention and early detection methods must be improved.^13^, ^14^ A further necessary aspect in the prevention would be the identification of risk factor profiles allowing to identify patients at high risk for foot disease.

A large number of articles have been published on this matter, however, with a large heterogeneity in the conducted studies and large differences in their quality. In contrast to more recent reviews on other aspects of the diabetic foot such as management and costs of this late complication,^15^, ^16^ only few reviews have been published on the associated risk factors, with the last publication in 2012.^17^ Both the presentation of results and the number of published articles since the last published review on risk factors for diabetic foot complications justify a most up‐to‐date systematic review, which was designed to identify and characterize the published risk factors associated with the DF in type 2 diabetes mellitus (T2DM), which comprises approximately 90%‐95% of all patients with diabetes.^18^ The results of the review should on the one hand guide physicians, researchers, patients and other interested parties in the identification of patients at high risk of developing DF complications and on the other hand identify risk factors that can serve as starting points to be tackled in order to reduce this risk.

## 2 MATERIALS AND METHODS

The protocol of this systematic review was developed according to the Preferred Reporting Items for Systematic Reviews and Meta‐Analyses: The PRISMA statement.^19^ To assure a comprehensive overview of the current literature, the databases MEDLINE, EMBASE, Cochrane, CINAHL, LISTA and Academic Search Elite were searched. The following approach was used: variations of terms for diabetes and also for foot or amputation or ulcer had to be included in the title of a publication, while, in addition, a variation of a term for risk or predictor had to be included in the abstract. The Boolean search term was chosen as follows: “(diab* OR T2DM):ti AND (foot OR amputation OR ulcer*):ti AND (risk* OR predict* OR determ* OR incidence):ab”.

The following inclusion and exclusion criteria were defined for the evaluation of the articles:

1. Only studies conducted in human subjects were included.
2. Only studies published in English language were included.
3. Diabetes and the outcome of interest (eg FU or LEA) had to be clearly defined.
4. The subject population had to consist of patients suffering from T2DM.
5. If the subject population was a mixed population with diabetes, the proportion of patients with T2DM had to be at least 75%.
6. The studies had to be at least of observational nature including a control group, that is patients with diabetes who developed foot complications had to be compared to patients with diabetes who did not.
7. Only studies on the first development of foot complications were included, which led to the exclusion of studies investigating recurrent complications or subsequent events after a first DF development.
8. To assure a minimum level of quality, the patient population had to consist of at least 100 subjects.
9. The risk factors had to be analysed in a multivariate model adjusted at least for age as a covariate.

**FIGURE 1:**
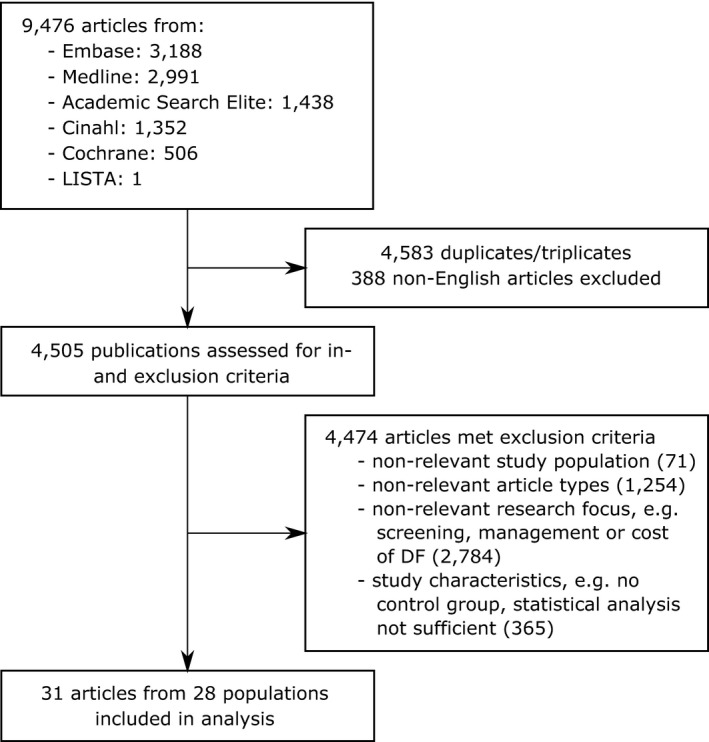
CONSORT diagram of literature search. Note: Indicated numbers for exclusion criteria represent the minimum number of articles. Articles were not evaluated for each criterion but were discarded as soon as one of the exclusion criteria was met

The search included publications published up until August 2019 when the database searches were performed. Repeating the search at time of submission in July 2020 identified no additional articles, which would warrant inclusion in this review. After removing duplicates and triplicates, all remaining publications were included in a screening of the abstracts and subsequently screening of the full articles. The initial screening was performed by the first author; ambiguous cases were discussed and decided with the corresponding author. In these steps, studies that did not fit the aforementioned inclusion and exclusion criteria were removed from further analysis (see Figure 1). The reference sections of included studies were checked in order to identify potential studies, which had been missed earlier and are relevant. Furthermore, if more than one publication analysed data from the same study or database, it was checked whether the subpopulations and/or risk factors differed between the publications, and only if this was the case, more than one publication was included from the same source of data. Otherwise, the most recent publication would have been included. After the final number of eligible studies has been identified, the publications were summarized in line with the approach published by Drinkwater et al, who performed a well‐structured, comprehensive, and easily understandable systematic review on risk factors for cataract in patients with T2DM.^20^ Due to the large clinical and methodological diversity of the included studies (concerning, eg patient populations, outcomes and study designs), the conduction of a systematic review was more reasonable than the performance of a meta‐analysis.^21^ Important characteristics and data from the eligible studies were brought together in tabular forms. The information entered included author and year of study, country, study design, study name, patient characteristics (sample size, number of events, baseline age at study entry, proportion of T2DM, proportion of female patients, diabetes duration at time of development of outcome, follow‐up time), potential conflicts of interest, methods and limitations, results from multivariate analyses as well as the covariates included in the models. The quality of included studies was assessed using the Newcastle‐Ottawa Quality Assessment Forms for Cohort Studies and Case‐Control Studies,^22^ with a median follow‐up time of 3 years chosen to be sufficient for outcome question 2 in case of cohort studies. The risk of bias was assessed for each included publication using the Cochrane handbook guidelines.^23^ In the following sections, for reporting effects for a specific potential risk factor we use the wording positive or negative association or relationship synonymously for statistically significant effects only. In addition, we use the notation consistent association if only positive effects and null effects or only negative effects and null effects have been reported and inconsistent association if both positive and negative effects have been reported.

## 3 RESULTS

Six databases were searched to retrieve all relevant literature on risk factors for the initial development of DF conditions. 9,476 publications were identified by predefined search terms. After removal of 4,583 duplicates and triplicates and 388 publications not written in English language, 4,505 references remained and were assessed for eligibility via screening of title, abstract and/or full text. A final number of 31 articles were included in the analysis (see Figure 1).^24^, ^25^, ^26^, ^27^, ^28^, ^29^, ^30^, ^31^, ^32^, ^33^, ^34^, ^35^, ^36^, ^37^, ^38^, ^39^, ^40^, ^41^, ^42^, ^43^, ^44^, ^45^, ^46^, ^47^, ^48^, ^49^, ^50^, ^51^, ^52^, ^53^, ^54^ The screening of the reference sections of these publications did not reveal any further articles meeting all specified inclusion and exclusion criteria, thus justifying the predefined search terms. The final sample comprised eleven cross‐sectional and twenty longitudinal studies. In the 31 articles, 28 different study populations were analysed, with two articles each from the Kaiser Permanente Northern Carolina Diabetes Registry (US),^30^, ^44^ the Diabetes Care in General Practice (DCGP) study (Denmark)^28^, ^29^ and the Taiwan National Health Insurance Research Database (NHIRD, Taiwan).^41^, ^45^ However, in all three cases, different subpopulations were included in the studies, and different risk factors were analysed in each of the publications. Therefore, all of the articles were considered for the systematic review. Associations between risk factors and the particular outcomes were given as the summary measures relative risk (RR), odds ratio (OR) or hazard ratio (HR).

The characteristics of all 31 articles, which were published between 1995 and 2019, are shown in Table 1. Six studies were performed in China,^32^, ^33^, ^36^, ^39^, ^51^, ^54^ five in the United States,^30^, ^34^, ^44^, ^49^, ^52^ three in Taiwan,^41^, ^45^, ^46^ two in the UK,^26^, ^35^ Denmark^28^, ^29^ and Saudi Arabia,^25^, ^37^ and one study each in Australia,^27^ Austria,^40^ Finland,^48^ Ghana,^43^ Italy,^47^ New Zealand,^42^ Pakistan,^53^ Republic of Nauru^38^ and Singapore.^50^ In addition, two multinational studies were included, one of which was conducted in Europe (UK, Switzerland, Germany, Poland, Croatia), East Asia (Hong Kong, Japan), the United States and Cuba,^31^ while the other one recruited subjects at sites across the UK, the United States and Canada.^24^ The sample sizes ranged from the lower bound for inclusion (100 subjects)^47^ up to more than 1.3 million subjects.^45^ While, in 17 studies, only subjects with T2DM were included, the proportion of subjects with T2DM in a mixed diabetic study population was at least 75% in eight studies. In six studies, the patient population was not further defined concerning the proportions of subjects with T1DM and T2DM. While, in most publications, the gender was distributed rather evenly, one study was performed on the US National Veterans Health Administration (VHA) database, in which the proportion of female patients was as low as 2.6%.^52^ The mean duration of diabetes ranged from 3.7 years^38^ to more than 13 years^25^, ^28^ in the different patient populations; however, this value was not stated in nine of the 31 articles.^24^, ^26^, ^34^, ^41^, ^45^, ^47^, ^48^, ^50^, ^52^ The mean follow‐up time in longitudinal studies varied between one year^24^, ^39^ and 13 years.^44^

**TABLE 1:** Characteristics of studies: Values for baseline age, diabetes duration and follow‐up time are given as mean, mean ± standard deviation or the range in parentheses, if not stated otherwise

Publication	Country	Study design	Study name	Sample size	Number of events	Baseline age (years)	T2DM (%)	Female (%)	Diabetes duration (years)	Follow‐up time (years)	Conflict of interest?
Abbott et al (1998)^24^	UK, USA, Canada	Retrospective cohort	No study name; RCT conducted by the ALCAR Foot Ulcer Study Group	1,035	109	60 (23‐70)	75.4	25.4	Not stated	1 for all subjects	Not stated
Al‐Rubeaan et al (2015)^25^	Saudi Arabia	Cross‐sectional (registry‐based) cohort	Saudi National Diabetes Registry (SNDR)	62,681	2,071	56.91 ± 13.54	95.45	47.6	13.29 ± 8.10	Not applicable (cross‐sectional)	None declared
Anderson et al (2018)^26^	UK	Retrospective cohort	No study name	13,955	1,147	69.4 (16‐89)	90.2	43.1	Not stated	Median: 10.5	None declared
Baba et al (2014)^27^	Australia	Prospective observational cohort	Fremantle Diabetes Study Phase 1 (FDS1)	1,292	16	64.0 ± 11.3	100	51.4	4.0 (IQR: 1.0‐9.0)	11.9 (0‐17.7)	None declared
Bruun et al (2013)^28^	Denmark	Prospective (registry‐based) cohort	Diabetes Care in General Practice (DCGP) study	1,381	88	65.4	100	46.9	At 6‐year follow‐up: 5.7 years at 14‐year follow‐up: 13.9 years	11.4	None declared
Bruun et al (2014)^29^	Denmark	Prospective (registry‐based) cohort	Diabetes Care in General Practice (DCGP) study	DF: 956 LEA: 1,058	DF: 28 LEA: 45	69.2	100	DF: 49.1 LEA: 48.7	5.7	Not stated	None declared
Callaghan et al (2011)^30^	USA	Prospective (registry‐based) cohort	Kaiser Permanente Northern California Diabetes Registry	28,701	981	59.4	96.4	46.1	Duration < 10 years: 64.6%	7.6	None declared
Chaturvedi et al (2001)^31^	Europe (UK, Switzerland, Germany, Poland, Croatia), East Asian (Hong Kong, Japan), American Indian (USA), Cuba	Prospective cohort	WHO Multinational Study of Vascular Disease in Diabetes	2,563	149	46.7	100	54.9	7.4	8.9 (for T1DM and T2DM, ie 3,443 subjects)	Not stated
Chen et al (2017)^32^	China	Cross‐sectional case‐control	No study name	1,269	578	63.8	100	44.8	9.5	Not applicable (cross‐sectional)	None declared
Chen et al (2018)^33^	China	Cross‐sectional case‐control	No study name	351	169	62.4	100	43.3	9.5	Not applicable (cross‐sectional)	Not stated
Dekker et al (2016)^34^	USA	Retrospective cohort	No study name	22,913	1,697	62 ± 14	Not stated	51.9	Not stated	Not stated	None declared
Hippisley‐Cox et al (2016)^35^	UK	Retrospective cohort	QResearch database	469,688	2,308	64.8	100	42.2	Newly diagnosed: 33.1% 1‐3 years: 24.6% 4‐6 years: 18.8% 7‐10 years: 13.2% >10 years: 10.3%	Not stated	Yes^a^
Hu et al (2012)^36^	China	Cross‐sectional case‐control	No study name	195	25	58.4	Not stated	43.1	7.3	Not applicable (cross‐sectional)	Not stated
Hu et al (2014)^37^	Saudi Arabia	Cross‐sectional case‐control	No study name	598	68	53.5	94.8	37.9	<5:26.7% 5‐10:23.3% 10.1‐20:31.6% >20:18.4%	Not applicable (cross‐sectional)	None declared
Humphrey et al (1996)^38^	Republic of Nauru (Central Pacific Ocean)	Retrospective cohort	No study name	375	46	46.5	100	54.8	3.7	Not stated	Not stated
Jiang et al (2015)^39^	China	Prospective cohort	No study name	At baseline: 1,333; at follow‐up after 1 year: 687	At baseline: 452; at follow‐up after 1 year: 229	58.7	100	41.1	8.7	687 patients followed up for 1 year	None declared
Kästenbauer et al (2001)^40^	Austria	Prospective cohort	No study name	187	10	58.6	100	45.5	10.5	3.6	Not stated
Lai et al (2015)^41^	Taiwan	Prospective cohort	Taiwan National Health Insurance Research Database (NHIRD)	45,087	1,588	56.2	100	46.1	Not stated	Not stated	None declared
Robinson et al (2016)^42^	New Zealand	Prospective cohort	New Zealand Diabetes Cohort Study	62,002	892	62.2	100	50	3.8	Median: 7.14	None declared
Sarfo‐Kantanka et al (2019)^43^	Ghana	Retrospective cohort	No study name given	3,143	78	55.9 ± 14.6	88.9	62.1	10.2 ± 5.6	Median: 4.2	None declared
Selby et al (1995)^44^	USA	Prospective case‐control	Kaiser Permanente Northern California Diabetes Registry	428	150	56.7	91.0	37	5.5	13.2	Not stated
Sheen et al (2018)^45^	Taiwan	Prospective cohort	Taiwan National Health Insurance Research Database (NHIRD)	1,307,723	9,738	64.4 ± 14.5	Not stated	36.1	Not stated	5	None declared
Tseng et al (2006)^46^	Taiwan	Cross‐sectional cohort	No study name	93,116	784	62.0 ± 11.6	96.5	53.9	7.3 ± 6.6	Not applicable (cross‐sectional)	None declared
Tuttolomondo et al (2017)^47^	Italy	Cross‐sectional case‐control	No study name	100	50	61.6	100	38	Not stated	Not applicable (cross‐sectional)	None declared
Venermo et al (2013)^48^	Finland	Retrospective (registry‐based) cohort	FinDM II database	In 1993:130,244 subjects; in 2007:274,388	Incidence in 1993:420 per 100,000 PY; incidence in 2007:154 per 100,000 PY	Not stated	Not stated	Not stated	Not stated	Not stated	None declared
Williams et al (2010 ^49^	USA	Prospective cohort	Pathways Epidemiologic Study	3,474	Not stated	64.1 ± 12.6	100	48	8.5 ± 8.2	4.1	None declared
Yang et al (2011)^50^	Singapore	Cross‐sectional cohort	No study name	44,917	1,457	65.0	Not stated	48.4	Not stated	Not applicable (cross‐sectional)	None declared
Ye et al (2014)^51^	China	Cross‐sectional cohort	No study name	829	61	56.0	100	42.3	5.7	Not applicable (cross‐sectional)	None declared
Young et al (2003)^52^	USA	Retrospective cohort	National Veterans Health Administration (VHA) database	429,918	11,794	64 ± 11	Not stated	2.6	Not stated	Not stated	Not stated
Younis et al (2018)^53^	Pakistan	Cross‐sectional cohort	No study name	1,940	144	51.24 ± 10.60	100	63	7.29 ± 6.1	Not applicable (cross‐sectional)	None declared
Zhao et al (2016)^54^	China	Cross‐sectional cohort	No study name	411	92	61.5	100	42.6	8.4	Not applicable (cross‐sectional)	None declared

Abbreviations: DF, diabetic foot; IQR, interquartile range; LEA, lower extremity amputation; PY, person‐years; RCT, randomized controlled trial; T1DM, type 1 diabetes mellitus; T2DM, type 2 diabetes mellitus; UK, United Kingdom; USA, United States of America; WHO, World Health Organization.

^a^ First author is codirector of QResearch and director of ClinRisk (a company that offers a software to implement clinical risk algorithms within clinical computer systems); the co‐author is a statistician at ClinRisk.

The methodological aspects and the corresponding limitations of the particular studies are summarized in Table 2. There were large variations concerning the definition of T2DM, ranging from criteria defined by the American Diabetes Association or the World Health Organization (WHO) to identification of patients with diabetes from charts or records via search for relevant diagnostic codes. In some publications, diabetes was assessed via self‐reported questionnaires. There were four main different outcomes: any diabetic foot (any DF), FU, LEA and Charcot arthropathy (CA). In some publications, also foot gangrene (FG) was assessed in addition.^25^, ^31^, ^37^ The outcomes were defined differently, ranging from WHO definition to individual classifications. The assessment of the outcome was in most cases performed via foot examination or via searches in medical records for relevant diagnostic procedure codes. The limitations of the included studies are discussed in Table 2. Most common limitations were missing patient characteristic data and the fact that cross‐sectional studies do not allow for the assessment of a causal relationship between risk factors and outcome. Furthermore, in many of the studies analysing LEA as end‐point, previous foot problems of patients have not been assessed. This did not allow a judgement on the novelty of foot conditions and assessment if initial development of foot conditions was evaluated.

**TABLE 2:** Methods and limitations of included studies

Publication	Methods	Measurement and definition of diabetes	Outcome	Measurement and definition of outcome	Limitations
Abbott et al (1998)^24^	Data from subjects of a discontinued RCT with 44 centres were analysed; diabetic patients aged 18‐70 y with PN, without PVD and without past or present FU were recruited	Based on WHO criteria	FU	FU defined as any full‐thickness skin lesion that required treatment in a hospital, with a GP or chiropodist; FU assessed by thorough foot examination	Only subjects with established PN included; duration of diabetes not specified; time to onset of first FU defined as number of days between study start and FU, not between date of diagnosis and FU
Al‐Rubeaan et al (2015)^25^	A population‐based register was used to identify diabetic patients aged ≥ 25 y from start of the register in 2000 until December 2012	Based on ADA criteria; diabetes glycaemic parameters collected from laboratory data according to patients' latest hospital visit	Any DF (FU, FG, LEA)	FU defined as current or history of nonhealing or poorly healing partial or full skin thickness wound below ankle; FG defined as tissue death and decay as results of ischaemia related to the foot, proven by Doppler study; LEA defined as minor distal or major proximal amputation related to diabetes	Only hospital‐based data in registry which limits generalizability of results; cross‐sectional study design limits ability to establish cause‐effect relationships between risk factors and foot complications
Anderson et al (2018)^26^	Diabetic patients aged 16‐89 y without a previous history of FU were recruited in 42 general practices between 01 January 2004 and 30 June 2015	Subjects were included from the database using relevant READ codes (coded thesaurus of clinical terms)	FU	Relevant READ codes were used to examine the health records of included individuals to identify the presence of FU with onset after 1 January 2004	Mean duration of diabetes not stated; variability in data entry of different GP practices; authors highlight potential for under‐reporting of FU
Baba et al (2014)^27^	Diabetic patients without active or past FU at baseline were recruited between 1993 and 1996 and followed up for hospitalization for FU until December 2010	Diabetes assessed based on clinical grounds and, if required, validation of case records	FU	All hospital admission for FU identified using ICD‐9 and ICD‐10 procedure codes, additional verification from case notes if required	Limiting the outcome to hospitalizations for FU may lead to a shift towards patients with more severe FUs; patient characteristics differ slightly between both outcomes (initial FU and FU during follow‐up), because different subpopulations were analysed
Bruun et al (2013)^28^	Subjects of both arms of a randomized trial newly diagnosed with diabetes between 1 March 1989 and 28 February 1992 and aged ≥ 40 y were recruited to the study and followed up for amputations until 01 January 2009 with follow‐up visits performed after 6 y (n = 970 subjects) and 14 y (n = 529 subjects)	Diagnosis based on hyperglycaemic symptoms and/or raised blood glucose values; confirmed with a single fasting whole blood/plasma glucose ≥ 7.0/8.0 mmol/l	FU, LEA	Outcome assessed by practitioners carrying out foot examinations; minor LEA defined as below the ankle and major LEA defined as through and above the ankle	The occurrence of FUs between the scheduled visits might not have been detected; assessment of FU not described
Bruun et al (2014)^29^	Subjects of both arms of a randomized trial newly diagnosed with diabetes between 01 March 1989 and 28 February 1992 and aged ≥ 40 y were recruited to the study, FUs analysed after 6 y (n = 956) and subjects followed up for amputation for 13 y	Diagnosis based on hyperglycaemic symptoms and/or raised blood glucose values; confirmed with a single fasting whole blood/plasma glucose ≥ 7.0/8.0 mmol/l	FU, LEA	Outcome assessed by practitioners carrying out foot examinations; minor LEA defined as below the ankle and major LEA defined as through and above the ankle	Mean follow‐up time not stated, assessment of FU not described
Callaghan et al (2011)^30^	Diabetic patients aged ≥ 19 y recruited for the study starting in 1995; follow‐up for identification of subjects with LEA was conducted until 31 December 2006	Diabetic patients identified from several sources including pharmacies (prescriptions for diabetic medications); laboratories (HbA1c > 6.7%) and outpatient, emergency room, and hospitalization records listing a diagnosis of diabetes	LEA	LEAs identified from discharge codes (via ICD‐9 procedure codes) and confirmed by chart review	Prior FU to the end‐point LEA not analysed
Chaturvedi et al (2001)^31^	Data from 10 of the original 14 centres of the study were analysed, diabetic subjects aged 35‐55 y were recruited, baseline examinations performed in 1975‐77 and follow‐up conducted until 01 January 1988	defined as patients under treatment for diabetes at a defined date	FG and/or LEA	Outcome assessed by questionnaire and examination; LEA and FG defined as past history of ischaemic gangrene, or an amputation of toe, foot or leg for arterial obstruction	Prior FU to the end‐point LEA not analysed
Chen et al (2017)^32^	Diabetic subjects were enrolled between January 2007 and March 2013	Based on 1999 WHO criteria	FU	FU defined according to 2015 IWGDF diagnostic criteria	Participants in FU group recruited from one hospital with rather severe courses of disease; therefore, generalizability of the results may be affected; cross‐sectional design does not allow the determination of a causal relationship between the potential risk factors and the outcome
Chen et al (2018)^33^	T2DM subjects with and without FU recruited between July 2013 and September 2015	Based on 2017 ADA criteria	FU	Based on 1981 Wagner classification system	Participants in DF group recruited from one hospital; therefore, generalizability of the results may be affected; cross‐sectional design does not allow the determination of a causal relationship between the potential risk factors and the outcome
Dekker et al (2016)^34^	Patients aged 18‐90 y with ≥ 3 documented HbA1c values over the period 2000‐2014 were added to the analysis	Diabetic patients identified via relevant ICD‐9 codes from electronic patient data	FU, CA	Patients with FU identified via relevant ICD‐9 codes from electronic patient data; most recent radiographic examination was examined for evidence of CA	Proportion of patients with T2DM not stated; mean duration of diabetes not stated; mean follow‐up time for subjects not stated
Hippisley‐Cox et al (2016)^35^	Primary care patients with T2DM aged 25‐84 y registered with eligible practices between 01 April 2007 and 31 January 2015 were identified and analysed	Diabetic patients identified via READ code for diabetes or more than one prescription for a hypoglycaemic drug	LEA	Identification of outcome via READ codes from primary care records, and via ICD‐10 codes and OPCS‐4 procedure codes from hospital and mortality records; LEA defined as including hindquarter, above knee or below knee amputations	Follow‐up time not stated; prior FU to the end‐point LEA not analysed
Hu et al (2012)^36^	Diabetic patients were recruited from February 2009 to October 2009, patients with impaired fasting glucose and impaired glucose tolerance were excluded	Patients previously diagnosed and treated for diabetes at the outpatient diabetic centre of the hospital in which the study was conducted	FU	Based on 2007 IWGDF diagnostic criteria	Proportion of patients with T2DM not stated; cross‐sectional design does not allow the determination of a causal relationship between the potential risk factors and the outcome
Hu et al (2014)^37^	Patients aged ≥ 30 y were recruited between June 2009 and May 2010 if they had been diagnosed with diabetes for at least 2 y; patients with current or past FU were excluded	Diagnosis self‐reported by patients and confirmed by physicians through medical chart records	Any DF (FU, FG, LEA)	DF complications reported by patients via questionnaire and confirmed by clinical examinations; patients were considered to have FU if they reported a history of FU or surgical debridement or have purulent discharge; patients were regarded as FG cases if they reported to have PAD history (colour change blackish); LEA was based on self‐reported toes or mid tarsal amputation or below‐ or above‐knee amputation	Cross‐sectional design does not allow the determination of a causal relationship between the potential risk factors and the outcome
Humphrey et al (1996)^38^	Data from diabetic patients aged ≥ 20 y were extracted from medical records and patients followed up for 12 y (1982‐1994) for first LEA during this time period	Based on 1985 WHO criteria	LEA	Minor LEA defined as any amputation distal to the ankle joint, major LEA defined as any amputation through or proximal to the ankle joint (definition according to the Global Lower Extremity Amputation Study); outcome was assessed by review of operating theatre records	Mean follow‐up time not stated; prior FU to the end‐point LEA not analysed
Jiang et al (2015)^39^	T2DM patients of eight hospitals recruited for one year starting on 01 June 2011; 687 subjects randomly selected for 1‐year follow‐up after the baseline visit	Based on 1999 WHO criteria	FU	FU defined as full‐thickness skin break at least of Wagner stage 1, occurring distal to the malleolus; details on past or present FU documented by examination and accessing the medical charts; cases defined as subjects who were admitted to hospitals for a diabetic FU	Only hospitalized patients analysed which reduces generalizability of results; short follow‐up period; follow‐up performed only via telephone interview; patients not seen by physicians after baseline; only 687 of 1,333 subjects followed up after 1 year
Kästenbauer et al (2001)^40^	T2DM patients ≤ 75 y without history of FU or other foot complications followed for development of FU (annual examination) from January 1994 to June 1998	Based on WHO criteria	FU	FU defined as full‐thickness neuropathic plantar or lateral forefoot ulceration penetrating the cutis and subcutis; FU assessed via thorough foot examination	Small number of subjects developed the outcome of interest (10 subjects developed FU)
Lai et al (2015)^41^	Data from diabetic patients collected between 01 January 2001 and 31 December 2010 were analysed; subjects with a history of LEA as well as subjects with a diabetes diagnosis prior to 01 January 2001 were excluded	Patients classified as diabetic if their records contained ≥ 3 outpatient diabetes codes within 365 calendar days	LEA	LEA identified via ICD‐9 procedure code for any LEA during hospitalization in the patients' inpatient records; minor amputation defined as any LEA distal to the ankle joint; major amputation defined as any LEA through or proximal to the ankle joint.	Mean duration of diabetes not stated; mean follow‐up time for subjects not stated; prior foot ulcerations to the end‐point LEA not analysed
Robinson et al (2016)^42^	Data from cohort of T2DM patients collected by primary care physicians between 2000 and 2006 were analysed	Diabetes determined by the patients' primary care physicians	LEA	Hospitalizations for LEA identified via the Australian version of ICD procedure codes (ICD‐9 and ICD‐10) from records of all stays in public hospitals in New Zealand	In Cox regression models, time from first recorded annual check to first LEA was used as time variable, not the time from diagnosis of diabetes to first LEA, therefore comparability to other studies limited; prior FU to the end‐point LEA not analysed
Sarfo‐Kantanka et al (2019)^43^	Patients were enrolled in a diabetes clinic of a hospital from 01 January 2010 to 31 December 2015 and followed up for LEAs (patients with FU or prior LEA were excluded at enrolment)	Based on 1998 WHO criteria	LEA	Minor LEA defined as amputation distal to the joint, major LEA defined as amputation through or proximal to the ankle joint; information retrieved from medical records and cross‐checked with charts of the hospital where the procedure was performed	Study conducted in a single hospital which limits the generalizability of the results
Selby et al (1995)^44^	Cases and controls selected from a cohort of diabetic patients between 1964 and 1984 (cases: diabetic subjects with first LEA after baseline, controls: diabetic subjects without LEA)	Patients were asked if they had been told by a doctor to have diabetes and/or if they currently use insulin or OHAs; diagnosis was confirmed by at least two abnormal glucose readings (fasting plasma glucose values > 140 mg/dL or postload or random values > 200 mg/dL) or one abnormal value plus initiation and continuation of insulin/OHA	LEA	LEAs identified by linking the diabetes cohort to hospital discharge files and identify LEA listing from 1971 to 1988 via relevant ICD‐8 or ICD‐9 codes; LEAs performed between 1964 and 1970 were identified by manual review of operation room logs	Prior FU to the end‐point LEA not analysed
Sheen et al (2018)^45^	Data from diabetic patients with LEA collected between 1998 and 2007 were retrieved from the database (patients diagnosed prior to 1997 were excluded); patients with LEA were compared to diabetic subjects without LEA	Patients enrolled with ≥ 1 hospital admission or ≥ 3 outpatient visits with a relevant diagnostic code within 365 calendar days (ICD‐9 or A codes), diagnosis confirmed via questionnaire	LEA	LEAs defined as amputations that occurred after diabetes diagnosis; patients with LEA were identified by linking the diabetic cohort to inpatient claims and identify subjects with LEA via relevant ICD‐9 codes.	Proportion of patients with T2DM not stated; mean duration of diabetes not stated; prior FU to the end‐point LEA not analysed
Tseng et al (2006)^46^	Diabetic patients ≥ 18 y seen in 66 hospitals and clinics located in Taiwan between 1995 and 1998 were interviewed by telephone between 1995 and 2002	Diagnosis of diabetes was assessed by relevant ICD‐9 codes	LEA	LEA defined by a self‐reported history of surgical resection of a part of the lower extremity on either side.	Prior FU to the end‐point LEA not analysed; cross‐sectional design does not allow the determination of a causal relationship between the potential risk factors and the outcome
Tuttolomondo et al (2017)^47^	Subjects with T2DM referred to a hospital between September 2014 and December 2015 were recruited in the study; cases were defined as diabetic subjects with FU; controls were defined as diabetic subjects without FU	Based on 2000 ADA criteria	FU	Based on WHO definition (DF defined as ulceration of the foot associated with PN and different grades of ischaemia); FU defined as a full‐thickness skin defect that required ≥ 14 days for healing	Mean diabetes duration not stated; cross‐sectional design does not allow the determination of a causal relationship between the potential risk factors and the outcome
Venermo et al (2013)^48^	All diabetic persons in Finland with any record in the national health and population registers from 1991 to 2007 were analysed and followed up for first LEA conducted from 1987 to 2007 (subjects with LEA were considered for the study only if they had a preceding 10‐year amputation‐free period)	A person was identified as having diabetes if he/she was on OHA (according to the national health insurance files) or he/she had been hospitalized for diabetes (according to the National Hospital Discharge Register)	LEA	All diabetic subjects were crosslinked with the National Hospital Discharge Register to identify patients with a LEA via relevant NOMESCO and Finish Hospital League procedure codes; LEA above the ankle was considered major, and LEA below the ankle was considered minor	Information on characteristics of study population was not retrievable; prior FU to the end‐point LEA not analysed; if preceding 10‐year period was amputation‐free, the patient is considered to have a first amputation; only risk factors for end‐point of major LEA assessed
Williams et al (2010)^49^	T2DM patients without prior FU or LEA recruited between 2000 and 2007; follow‐up was performed 5 y later	Patients considered diabetic if any of the following in the preceding 12 months was applicable: dispensed prescription for insulin or an OHA; 2 fasting plasma glucose levels ≥ 126 mg/dL; 2 random plasma glucose levels ≥ 200 mg/dL; or 2 outpatient diagnoses of diabetes or any inpatient diagnosis of diabetes	FU	Patients were screened for FU via ICD‐9 codes, and diagnosis was confirmed by chart review; FU defined as a break in the skin extending through the dermis to deeper tissue in a location distal to the medial and lateral malleoli that had not healed in 30 days	Large number of subjects lost to follow‐up
Yang et al (2011)^50^	Diabetic patients aged ≥ 18 y admitted to a single hospital between 01 January 2004 and 31 December 2009 identified in hospital discharge database and included in the study	Diabetes defined by Australian version of ICD‐9 codes	LEA	LEAs identified from inpatient records via the Australian version of ICD‐9 diagnostic and procedure codes	Proportion of patients with T2DM not stated; mean duration of diabetes not stated; prior FU to the end‐point LEA not analysed; cross‐sectional design does not allow the determination of a causal relationship between the potential risk factors and the outcome
Ye et al (2014)^51^	T2DM patients who visited a diabetes clinic between January 2007 and December 2009 were included in the study	Diabetes status assessed including self‐reported diabetes and newly diagnosed diabetes based on 1999 WHO criteria; patients had to be either on OHA or insulin at the time of recruitment	FU	FU defined as a nonhealing or poorly healing partial or full‐thickness wound below the ankle in an individual with diabetes	Multivariate analysis only performed in female subjects; cross‐sectional design does not allow the determination of a causal relationship between the potential risk factors and the outcome
Young et al (2003)^52^	Diabetic subjects who received primary care within the US Veterans Affairs Health Care System between 01 October 1997 and 30 September 1998 were selected for the study; ≥3 visits for that year were needed for the patients to be included in the study	Diagnosis of diabetes assessed via the presence of at least one outpatient or inpatient visit with a relevant ICD‐9 diagnosis code	LEA	LEAs identified via relevant ICD‐9 procedure codes	Proportion of patients with T2DM not stated; mean duration of diabetes not stated, mean duration of follow‐up time not stated; prior FU to the end‐point LEA not analysed; results were obtained from a veteran population which might limit the generalizability of the results
Younis et al (2018)^53^	T2DM patients aged ≥ 30 y recruited from a hospital between January 2016 and January 2017	Diabetic condition verified via review of medical records and previous laboratory tests	FU	FU assessed via complete foot examination	Cross‐sectional design does not allow the determination of a causal relationship between the potential risk factors and the outcome
Zhao et al (2016)^54^	T2DM subjects admitted to a hospital between October 2011 and September 2012 were enrolled in the study	Based on 2010 ADA criteria	FU	Based on WHO definition: FU defined as ulceration of the foot (distally from the ankle and including the ankle) associated with PN and different grades of ischaemia and infection	Cross‐sectional design does not allow the determination of a causal relationship between the potential risk factors and the outcome

Abbreviations: ADA, American Diabetes Criteria; CA, Charcot arthropathy; DF, diabetic foot; FG, foot gangrene; FU, foot ulceration; GP, general practitioner; ICD, International Statistical Classification of Diseases and Related Health Problems; IWGDF, International Working Group on the Diabetic Foot: LEA, lower extremity amputation; NOMESCO, Nordic Medico‐Statistical Committee; OHA, oral hypoglycaemic agent; OPCS, Office of Population Censuses and Surveys; PAD, peripheral arterial disease; PN, peripheral neuropathy; PVD, peripheral vascular disease; RCT, randomized controlled trial; T2DM, type 2 diabetes mellitus; WHO, World Health Organization.

Table 3 shows the results of the individual studies including the published summary measures, and—if stated—the corresponding confidence intervals and p‐values. In addition to the results of the multivariate analyses, the covariates included in the analyses are listed.

**TABLE 3:** Outcomes and results of included studies

Publication	Outcome	Results in multivariate analyses	Covariates
Abbott et al (1998)^24^	FU	stat. sign.: age (HR 0.957), PN (1.050), VPT (1.056); not stat. sign.: type of diabetes, ethnicity, economic status, duration of diabetes	Age, PN, VPT, type of diabetes, ethnicity, economic status, duration of diabetes
Al‐Rubeaan et al (2015)^25^	Any DF (FU, FG, LEA)	stat. sign.: age (≥45 y: OR 3.81 [95% CI: 2.22‐6.54], *P* < .0001), male gender (1.92 [1.49‐2.48], *P* < .0001), PN (7.20 [4.84‐10.71], *P* < .0001), duration of diabetes (≥10 y: 2.50 [1.66‐3.77], *P* < .0001), insulin use (3.98 [3.02‐5.23], *P* < .0001), retinopathy (1.84 [1.43‐2.35], *P* < .0001), poor glycaemic control (1.49 [1.12‐1.98], *P* = .006); not stat. sign.: Charcot joint, PVD, nephropathy, cerebral vascular disease, coronary heart disease, hypertension, smoking	Age, gender, Charcot joint, PVD, PN, duration of diabetes, insulin use, retinopathy, nephropathy, glycaemic control, cerebral vascular disease, coronary heart disease, hypertension, smoking
Anderson et al (2018)^26^	FU	stat. sign.: social deprivation (highest quintile of deprivation compared to lowest quintile) (OR 1.77 [95% CI: 1.45‐2.14], *P* < .0001) in T2DM only: increased deprivation per quintile (1.13 [1.09‐1.16], *P* < .0001); not stat. sign.: increased deprivation per quintile in patients with T1DM	Age, gender, social deprivation
Baba et al (2014)^27^	FU	Risk factors for active FU at baseline: stat. sign.: intermittent claudication (OR 17.24 [95% CI 3.66‐81.23), *P* < .001), duration of diabetes (per increase of 5 y: 1.58 [1.12‐2.23], *P* = .009), PN (15.84 [1.95‐128.81], *P* = .010), antihypertensive therapy (11.16 [1.13‐95.44], *P* = .028) not stat. sign.: age, exercise, diabetes treatment, microalbuminuria, PVD, history of vascular bypass Risk factors for hospitalization for FU during follow‐up: stat. significant: retinopathy (OR 3.86 [95% CI 2.26‐6.59], *P* < .001), cerebrovascular disease (3.76 [1.97‐7.19], *P* < .001), intermittent claudication (2.77 [1.52‐5.04], *P* = .001), PN (2.24 [1.35‐3.71), *P* = .002), HbA1c (for a 1% increase: 1.22 [1.07‐1.40], *P* = .003), alcohol consumption (for 1 standard drink/day increase: 1.16 [1.05‐1.27], *P* = .003), decreased eGFR (2.12 [1.30‐3.51], *P* = .004), PVD (1.85 [1.10‐3.13], *P* = .021), pulse pressure (for a 5 mmHg increase: 1.07 [1.00‐1.14], *P* = .038); not stat. sign.: duration of diabetes, fasting plasma glucose, diabetes treatment, systolic blood pressure, albuminuria, history of vascular bypass	For end‐point active ulcer at baseline: age, exercise, duration of diabetes, diabetes treatment, antihypertensive therapy, PN, intermittent claudication, PVD, history of vascular bypass For end‐point hospitalization for FU during follow‐up: duration of diabetes, alcohol consumption, fasting plasma glucose, HbA1c, diabetes treatment, systolic blood pressure, pulse pressure, albuminuria, nephropathy, retinopathy, PN, intermittent claudication, PVD, cerebrovascular disease, history of vascular bypass
Bruun et al (2013)^28^	FU, LEA	Risk factors for FU at baseline: stat. sign.: male gender (OR 2.45 [95% CI 1.01‐5.98], *P* < .05), PN (2.51 [1.30‐4.85], *P* < .01), retinopathy (6.21 [2.13‐18.10], *P* < .001), PVD (3.22 [1.46‐7.13], *P* < .01); not stat. sign.: age, impaired vision or blindness, microalbuminuria, proteinuria, stroke, myocardial infarction, angina/ischaemic heart disease, mental disorder Risk factors for FU at 6‐year follow‐up: stat. sign.: PN (2.72 [1.24‐5.96], *P* < .05), PVD (2.84 [1.10‐7.37], *P* < .05), myocardial infarction (4.36 [1.60‐11.91], *P* < .01); not stat. sign.: age, gender, retinopathy, impaired vision or blindness, microalbuminuria, proteinuria, stroke, angina/ischaemic heart disease, heart failure, cancer, mental disorder Risk factors for FU at 14‐year follow‐up: stat. sign.: PN (5.60 [1.98‐15.88], P <.01), PVD (5.15 [1.59‐16.74], *P* < .01), myocardial infarction (3.40 [1.07‐10.81], *P* < .05), heart failure (4.76 [1.40‐16.15], *P* < .05); not stat. sign.: age, gender, retinopathy, impaired vision or blindness, microalbuminuria, proteinuria, stroke, angina/ischaemic heart disease, mental disorder Risk factors for any amputation during follow‐up: male gender (HR 2.40 [95% CI 1.31‐4.41], *P* < .01), PN (2.09 [1.19‐3.69], *P* < .05), retinopathy (6.42 [2.59‐15.90], *P* < .001), impaired vision or blindness (6.92 [2.35‐20.38], *P* < .001), microalbuminuria (2.11 [1.21‐3.67], *P* < .01), PVD (3.43 [1.65‐7.12], *P* < .001), myocardial infarction (2.79 [1.01‐7.75], *P* < .05); not stat. sign.: age, proteinuria, stroke, angina/ischaemic heart disease, heart failure, cancer, mental disorder	Age, gender, duration of diabetes, living alone, education, smoking, HbA1c, BMI, hypertension, PN, retinopathy, impaired vision or blindness, microalbuminuria, proteinuria, PVD, stroke, myocardial infarction, angina/ischaemic heart disease, heart failure, cancer, mental disorder
Bruun et al (2014)^29^	FU, LEA	Risk factors for FU at 6‐year follow‐up: stat. sign.: patient's motivation reported by GP (poor vs very good: OR 12.37 [95% CI 1.22‐25.23], *P* < .05), patient's own effort reported by GP (poor vs good: 6.24 [2.16‐18.01], *P* < .05); not stat. sign.: patient‐reported effort, influence of life circumstances as reported by GP Risk factors for any amputation during 13‐year follow‐up: stat. sign.: patient's own effort reported by GP (poor vs good: HR 4.17 [95% CI 1.67‐10.45], *P* < .01), life circumstances as reported by GP (none in particular vs good: 2.96 [1.07‐8.22], *P* < .05; poor vs good: 2.60 [1.03‐6.54], *P* < .05); not stat. sign.: patient's motivation reported by GP, patient‐reported effort	Age, gender, duration of diabetes, living alone, education, smoking, HbA1c, BMI and hypertension, patient's motivation reported by GP, patient's effort reported by GP, patient‐reported effort, influence of life circumstances as reported by GP
Callaghan et al (2011)^30^	LEA	stat. sign.: triglycerides (150‐199 vs < 150 mg/dL: HR 1.29 [95% CI 1.07‐1.55]; 200‐499 vs < 150 mg/dL: 1.40 [1.19‐1.65]; >500 vs < 150 mg/dL: 1.65 [1.22‐2.24]), LDL (>160 vs < 100 mg/dL: 1.30 [1.03‐1.64]), HDL (>60 vs < 40 mg/dL: 1.37 [1.02‐1.84]), male gender (1.59 [1.33‐1.90]), ethnicity (Asian vs white: 0.51 [0.39‐0.69]), duration of diabetes (10‐19 vs < 10 y: 1.94 [1.65‐2.28], >20 vs < 10 y: 2.38 [1.96‐2.88]), diabetes therapy (T2DM on insulin vs diet only: 2.41 [1.88‐3.10], T2DM on oral OHA vs diet only: 1.62 [1.28‐2.05]), BMI (obese vs normal weight: 0.80 [0.65‐0.98]), height (2nd vs 1st quartile: 1.43 [1.09‐1.86], 3rd vs 1st quartile: 1.34 [1.01‐1.77], 4th vs 1st quartile: 1.98 [1.48‐2.66]), hypertension (1.51 [1.27‐1.78]), PN (2.60 [2.23‐3.04]), retinopathy (1.85 [1.15‐2.98]), heart attack (1.27 [1.06‐1.52]), stroke (1.97 [1.55‐2.50]), end‐stage renal disease (4.29 [3.06‐6.03]); not stat. sign.: LDL (100‐129 and 130‐159 both vs < 100 mg/dL), HDL (40‐59 vs < 40 mg/dL), age, ethnicity (African American, Hispanic, Mixed/Other, all vs White), HbA1C, statin medication, fibrate/niacin medication, smoking, BMI (underweight and overweight, both vs normal weight)	Age, gender, ethnicity, triglycerides, LDL, HDL, education, income, whether lives in working class neighbourhood, smoking, alcohol use, BMI, height, adherence to guidelines for self‐monitoring of blood glucose, exercises, statin medication, fibrate/niacin medication, family history of diabetes, duration of diabetes, HbA1C, type of diabetes and therapy, history of hypertension, neuropathy, retinopathy, nephropathy, stroke or heart attack, end‐stage renal disease
Chaturvedi et al (2001)^31^	FG and/or LEA	stat. sign.: ethnicity (American Indian vs European: RR 2.78 [95% CI 1.66‐4.66])	Age, duration of diabetes, gender, ethnicity, ECG, plasma glucose, systolic blood pressure, proteinuria, retinopathy, triglyceride
Chen et al (2017)^32^	FU	stat. sign.: indirect bilirubin (≥ 6 μmol/l vs < 6 μmol/l: OR 0.75 [95% CI 0.57‐0.98], *P* = .029); not stat. sign.: total bilirubin, direct bilirubin	Age, gender, smoking, alcohol, BMI, HbA1C, WBC, ALT, AST, GGT, triglycerides; model for analysis of direct bilirubin in addition adjusted for indirect bilirubin, and vice versa
Chen et al (2018)^33^	FU	stat. sign.: VEGF‐A (lower 1st tertile vs upper 3rd tertile: OR 1.76 [95% CI 1.01‐3.07], analysed as continuous variable per 10‐unit increase: 0.93 [0.88‐0.97]), PlGF (lower 1st tertile 1 vs upper 3rd tertile: 2.36 [1.34‐4.15], analysed as continuous variable per 5‐unit increase: 0.96 [0.94‐0.99]); not stat. sign.: VEGF‐A (middle 2nd tertile vs upper 3rd tertile), PlGF (middle 2nd tertile vs upper 3rd tertile)	Age, gender, duration of diabetes, education, BMI and smoking, VEGF‐A, PlGF
Dekker et al (2016)^34^	FU, CA	Risk factors for FU: stat. sign.: age (for every year increase: OR 0.991 [95% CI 0.985‐0.997], *P* = .003), retinopathy (1.357 [1.154‐1.595], *P* < .001), PN (3.441 [2.94‐4.027], *P* < .001), hypertension (2.265 [1.586‐3.237], *P* < .001), PVD (4.309 [3.668‐5.062], *P* < .001), coronary artery disease (1.388 [1.178‐1.635], *P* << .001], chronic kidney disease (1.824 [1.541‐2.158], *P* < .001); not stat. sign.: number of HbA1cs drawn, most recent BMI Risk factors associated with CA: stat. sign.: age (for every year increase: 0.964 [0.938‐0.99], *P* = .008), hypertension (2.571 [1.213‐4.131], *P* = .018), PN (1.233 [1.035‐3.038], *P* = .049); not stat. sign.: number of HbA1cs drawn, most recent BMI, retinopathy, PVD, coronary artery disease, chronic kidney disease	Age, number of HbA1Cs drawn BMI, retinopathy, neuropathy, hypertension, PVD, coronary artery disease, chronic kidney disease
Hippisley‐Cox et al (2016)^35^	LEA	stat. sign.: metformin (HR 0.70 [95% CI 0.64‐0.77]), insulin (1.64 [1.41‐1.91]) (HR for each diabetes drug group is compared with no prescription of that particular medicine); not stat. sign.: glitazones, gliptins, sulphonylureas, other OHA	Age, gender, ethnicity, calendar year, duration of diabetes, deprivation, smoking, use of anticoagulants, thiazides, ACE inhibitors, angiotensin 2 blockers, calcium channel blockers, statins, aspirin, blindness, hyperglycaemia, hypoglycaemia, severe kidney failure, hypertension, CVD, atrial fibrillation, nephropathy, rheumatoid arthritis, valvular heart disease, PVD, BMI, systolic blood pressure, HbA1c, creatinine, cholesterol:HDL ratio, each of the other diabetes drugs
Hu et al (2012)^36^	FU	stat. sign.: skin autofluorescence (OR 2.55 [95% CI 1.10‐5.91], *P* = .03), triglycerides (0.31 [0.13‐0.74], *P* < .01), BUN (1.22 [1.02‐1.46], *P* = .03), right ABI (0.001 [0.000‐0.04], *P* < .01), C‐reactive protein (1.02 [1.001‐1.03], *P* = .03); not stat. sign.: duration of diabetes, age, left ABI, HDL, creatinine, LDL, VPT	Age, duration of diabetes, skin autofluorescence, BUN, creatinine, triglyceride, HDL, LDL, C‐reactive protein, left ABI, right ABI, VPT
Hu et al (2014)^37^	Any DF (FU, FG, LEA)	risk factors associated with FU, FG and/or LEA: nationality (non‐Saudi vs Saudi: OR 2.47 [95% CI 1.39‐4.38], *P* = .002), PN (3.21 [1.69‐6.10], *P* < .0001), PVD (2.80 [1.56‐5.01], *P* < .001), duration of diabetes (10.1‐20 y vs < 5 y: 3.70 [1.26‐10.84]; >20 y vs < 5 y: 3.60 [1.09‐11.89]); not stat. sign.: gender, age, inulin use, OHA use, clopidogrel use, duration of diabetes (5‐10 y vs < 5 y), haemoglobin (125‐138 g/l, 138‐149 g/l, ≥149 g/l, all vs < 125 g/l)	Multivariate logistic regression model adjusted for: age, gender, nationality, insulin use, OHA use, clopidogrel use, duration of diabetes, haemoglobin, PN, PVD
Humphrey et al (1996)^38^	LEA	stat. sign.: fasting plasma glucose (per 1mmol/l increment: RR 1.26 [95% CI 1.14‐1.38], *P* < .001), diabetes duration (per year increase: 1.15 [1.07‐1.23], *P* < .001), female gender (0.34 [0.18‐0.83], *P* = .015), systolic blood pressure (per 10 mmHg: 0.78 [0.76‐0.80], *P* = .010); not stat. sign.: age, BMI, total plasma cholesterol, fasting plasma triglycerides, mean daily alcohol intake, smoking	Age, gender, duration of diabetes, mean daily alcohol intake, smoking, BMI, systolic blood pressure, total plasma cholesterol, fasting plasma triglycerides, fasting plasma glucose
Jiang et al (2015)^39^	FU	Risk factors associated with FU at baseline: stat. sign.: male gender (OR 2.062 [95% CI 1.323‐3.215], *P* = .001), smoking (1.597 [1.057‐2.411], *P* = .026), location (city vs rural: 2.234 [1.515‐3.293], *P* < .0001), retinopathy (1.781 [1.234‐2.569], *P* = .002), ABI < 0.9 (5.452 [3.489‐8.519], *P* < .0001), intermittent claudication (5.216 [2.763‐9.848), *P* < .0001), diabetes therapy (insulin vs OHA: 4.205 [2.247‐7.869], *P* < .0001; OHA and insulin vs OHA: 2.526 [1.323‐4.824], *P* = .005), BMI (0.927 [0.883‐0.927], *P* = .002), HDL (per unit increase: 0.238 [0.134‐0.423], *P* < .0001), haemoglobin (per unit increase: 0.976 [0.970‐0.985], *P* < .0001), postprandial blood glucose (0.940 [0.908‐0.972], *P* < .0001); not stat. sign.: age, living alone (yes/no), occupation, hypertension, PN, PVD, nephropathy, cataracts, duration of diabetes, HbA1c, fasting plasma glucose, bilirubin, creatinine, cholesterol, triglyceride, albumin, WBC Risk factors associated with FU at follow‐up: stat. sign.: HDL (OR 0.427 [95% CI 0.228‐0.799], *P* = .008), nephropathy (2.320 [1.449‐3.714], *P* < .0001), diabetes therapy (insulin vs OHA: 3.136 [1.357‐7.251], *P* = .008; OHA and insulin vs OHA: 2.629 [1.125‐6.148], *P* = .026); not stat. sign.: all other factors also analysed at baseline	Age, gender, location, living alone, occupation, smoking, hypertension, PN, PVD, nephropathy, retinopathy, cataracts, duration of diabetes, diabetes therapy, ABI, intermittent claudication, BMI, HbA1c, fasting plasma glucose, postprandial blood glucose, bilirubin, creatinine, cholesterol, triglyceride, HDL, haemoglobin, albumin, WBC
Kästenbauer et al (2001)^40^	FU	stat. sign.: elevated VPT (RR 25.4 [95% CI 3.1‐205], *P* = .0024), mean plantar pressure (6.3 [1.2‐32.7], *P* = .0291), daily alcohol intake (5.1 [1.1‐24.0], *P* = .0404), mediasclerosis (0.07 [0.01‐0.6], *P* = .0174); not stat. sign.: age, diabetes duration, body weight, OHA therapy, insulin use, history of angiography, flatfoot deformity, hallux valgus, oxford shoes, varicosis, dry skin, skeletal abnormalities, HbA1c, triglycerides, stage of peroneal nerve conduction velocity, diastolic blood pressure	Age, elevated VPT, elevated mean plantar pressure, diabetes duration, body weight, OHA therapy, insulin use, history of angiography, daily alcohol intake, flatfoot deformity, hallux valgus, oxford shoes, varicosis, dry skin, mediasclerosis, skeletal abnormalities, HbA1c, triglycerides, stage of peroneal nerve conduction velocity, diastolic blood pressure
Lai et al (2015)^41^	LEA	stat. sign.: age at T2DM onset (HR 1.024 [95% CI 1.013‐1.035]), male gender (1.643 [1.237‐2.183]), heart failure (2.134 [1.445‐3.151]), hypertension (0.674 [0.496‐0.915]), coronary artery disease (0.705 [0.502‐0.988]), hyperlipidaemia (0.361 [0.269‐0.486]), retinopathy (2.067 [1.118‐3.821]), PN (2.338 [1.617‐3.38]), peripheral arterial occlusive disease (4.134 [2.717‐6.289]); not stat. sign.: chronic kidney disease, atrial fibrillation, stroke, nephropathy	Age, gender, heart failure, chronic kidney disease, hypertension, coronary artery disease, hyperlipidaemia, atrial fibrillation, stroke, nephropathy, retinopathy, PN, peripheral arterial occlusive disease
Robinson et al (2016)^42^	LEA	stat. sign.: ethnicity (East Asian vs European/other: HR 0.23 [95% CI 0.10‐0.56], *P* < .001; Indian vs European/other: 0.48 [0.27‐0.83], *P* < .001; Maori vs European/other: 1.61 [1.35‐1.93], *P* < .001), age at onset (per 10 y: 1.52 [1.42‐1.63], *P* < .001), female gender (0.72 [0.60‐0.87], *P* < .001), diabetes duration (per year: 1.19 [1.17‐1.22], *P* < .001), smoking status (ex‐smoker vs nonsmoker: 1.26 [1.09‐1.47], *P* = .003; current smoker vs nonsmoker: 1.63 [1.35‐1.97], *P* < .001), height (per 10 cm: 1.35 [1.23‐1.48], *P* < .001), systolic BP (per 10 mmHg: 0.69 [0.53‐0.89], *P* = .005; squared: 1.01 [1.01‐1.02], *P* = .001), HbA1c (per 10 mmol/mol: 1.27 [1.24‐1.31], *P* < .001), total/HDL‐cholesterol ratio (1.05 [1.02‐1.09], *P* = .007); not stat. sign.: ethnicity (Pacific vs European/other), weight, BMI	Age, gender, ethnicity, diabetes duration, smoking status, height, systolic BP, HbA1c, total/HDL‐cholesterol ratio, weight, BMI
Sarfo‐Kantanka et al (2019)^43^	LEA	stat. sign.: age (per 10‐year increase: HR 1.11 [95% CI 1.06‐1.22], *P* < .001), male gender (3.50 [2.88‐5.23], *P* < .001), type of diabetes (T2DM vs T1DM: 8.21 [2.58‐1.07], *P* < .001), BMI (each 5kg/m^2^ increase: 3.2 [2.51‐7.25], *P* < .001), HbA1c (per % increase: 1.11 [1.05‐1.25], *P* = .03), hypertension (1.14 [1.12‐3.21], *P* < .001), PN (6.56 [6.21‐8.52], *P* < .001), PVD (7.73 [4.39‐9.53], *P* < .001); not stat. sign.: duration of diabetes, dyslipidaemia, nephropathy	Variables included in the model were as follows: age, gender, duration of diabetes, type of diabetes, BMI, glycaemic control (HbA1c), lipid status, hypertension, renal function, PN, PVD
Selby et al (1995)^44^	LEA	stat. sign.: glucose score (OR 1.75 [1.37‐2.24]), systolic blood pressure (per 1 mm Hg: 1.02 [1.01‐1.04]), retinopathy (3.68 [1.78‐7.62]), PN (4.05 [2.01‐8.17]), stroke (2.70 [1.27‐5.75]); not stat. sign.: duration of diabetes, type of diabetes, BMI, treatment (insulin and OHA, both vs diet only), ethnicity (black and other, both vs white), total cholesterol, smoking status (never or ex‐smoker vs current smoker), myocardial infarction	Age, gender, glucose score, duration of diabetes, type of diabetes, BMI, treatment, ethnicity, systolic blood pressure, total cholesterol, smoking, retinopathy, PN, stroke, myocardial infarction
Sheen et al (2018)^45^	LEA	stat. sign.: age (5 age groups [35‐45, 45‐55, 55‐65, 65‐75, >75 y] compared to < 35 y: each HR ≥ 1.73, each *P* < .0001), male gender (HR 1.83 [95% CI 1.756‐1.916], *P* < .0001), salary (8 salary groups [insured dependents, ≤15,840; 15,841‐22,800; 22,801‐28,800; 28,801‐36,300; 36,301‐45,800; 45,801‐57,800; 57,801‐72,800] compared to > 72,801: each HR ≥ 4.67, each *P* < .0009), low income status (3.69 [3.387‐4.028), *P* < .0001), diabetic complications (different number of complications [1, 2, 3, 4, ≥5] compared to no complications: each HR ≥ 1.68, each *P* < .0001, city household income (middle vs high: 1.12 [1.066‐1.178], *P* < .0001), degree of urbanization (urbanization divided into 8 levels; all levels compared with highest level of urbanization: each HR ≥ 1.26; each *P* < .0001), attending clinic for regular care is not a metabolic disease clinic (1.47 [1.362‐1.591], *P* < .0001), ownership of hospital for regular care (nonprofit vs public: 1.16 [1.085‐1.248], *P* < .0001), not attending preventive programme ‘P4P Care’ (3.46 [3.187‐3.758], *P* < .0001); not stat. sign.: household income (low vs high), ownership of hospital for regular care (private vs public)	Age, gender, salary, income status, number of diabetic complications, city household income, degree of urbanization, metabolic disease clinic (for patient's regular care), ownership of hospital (for patient's regular care), attendance of preventive programme ‘P4P Care’
Tseng et al (2006)^46^	LEA	stat. sign.: age (10‐year increment: OR 1.19 [95% CI 1.10‐1.28], *P* < .01), type of diabetes (1.67 [1.24‐2.25), *P* < .01), duration of diabetes (10‐year increment: 1.78 [1.65‐1.93], *P* < .01), smoking status (ex‐smoker vs never smoker: 1.33[1.05‐1.69], *P* < .05), hypertension (1.34 [1.15‐1.57], *P* < .01), body height (10‐cm increment: 1.16 [1.03‐1.32], *P* < .05); stat. sign. risk factors studied in subset of 9,295 subjects: fasting plasma glucose (0.6 mmol/l increment: 1.12 [1.04‐1.21], *P* < .01); not stat. sign.: gender, smoking status (current vs never smoked), dyslipidaemia (yes vs no; and unknown vs no)	Age, gender, duration of diabetes, type of diabetes, smoking, hypertension, height, fasting plasma glucose, dyslipidaemia
Tuttolomondo et al (2017)^47^	FU	stat. sign.: hypertension (OR 21.27 [95% CI 4.09‐110.62], *P* = .0001), dyslipidaemia (6.07 [1.43‐25.66], *P* = .014), BMI (1.17 [1.02‐1.34], *P* = .019), pulse wave velocity (2.26 [1.36‐3.75], *P* = .002), reactive hyperaemia index (0.01 [0.001‐0.185], *P* = .002); not stat. sign.: age, systolic blood pressure, aortic augmentation index, cognitive function (Mini‐Mental State Examination)	Age, hypertension, dyslipidaemia, BMI, systolic blood pressure, arterial stiffness (aortic augmentation index, pulse wave velocity), endothelial function (reactive hyperaemia index), cognitive function (Mini‐Mental State Examination)
Venermo et al (2013)^48^	LEA	stat. sign.: age (4 age groups [50‐64, 65‐74, 75‐84, >85 y] all compared to 30‐39 y: each HR ≥ 3.07, each *P* < .0001), socio‐economic position (4 quintiles [2nd, 3rd, 4th and 5th = highest quintile] compared with 1 = lowest quintile: each HR ≤ 0.89; each *P* < .001), female gender (HR 0.62 [95% CI 0.59‐0.65], *P* < .001), type of diabetes (T2DM vs T1DM: 0.57 [0.54‐0.61], *P* < .001), diabetes duration (10‐19 y vs 0‐9 y: 2.50 [2.36‐2.64], *P* < .001; ≥20 y vs 0‐9 y: 3.30 [3.09‐3.52], *P* < .001), amputation year (per year from 1987 to 2007:0.93 [0.92‐0.93], *P* < .001)	Age, gender, socio‐economic position, diabetes type, duration of diabetes, year of amputation
Williams et al (2010)^49^	FU	stat. sign.: major depression compared to no depression (HR 2.00 [95% CI 1.24‐3.25]); not stat. sign.: minor depression compared to no depression	Age, gender, ethnicity, education, marital status, diabetes duration, insulin use, number of diabetes complications, BMI, smoking status, foot self‐care, HbA1c
Yang et al (2011)^50^	LEA	stat. sign.: age ≥ 65 (OR 0.8 [95% CI 0.71‐0.89], *P* < .001), female gender (0.79 [0.71‐0.87], *P* < .001), year of discharge (2007 vs 2004:0.72 [0.60‐0.87], *P* = .001; 2008 vs 2004:0.58 [0.48‐0.70], *P* < .001; 2009 vs 2004:0.40 [0.34‐0.49], *P* < .001), ethnicity (Malay vs Chinese: 1.55 [1.35‐1.77], *P* < .001), renal disease (3.18 [2.84‐3.56], *P* < .001); not stat. sign.: year of discharge (2005 vs 2004; 2006 vs 2004), ethnicity (India vs Chinese; Other vs Chinese)	Age, gender, ethnicity, year of discharge, nephropathy
Ye et al (2014)^51^	FU	stat. sign. in female patients: uric acid (for every 1‐μmol/L increment: OR 1.004 [95% CI 1.001‐1.008], *P* < .05; quintile 5 vs quintile 1:4.727 [1.357‐16.468], *P* < .05); not stat. sign.: uric acid (quintiles 2, 3, 4, each vs quintile 1 [lowest concentration of uric acid])	Age, duration of diabetes, uric acid, PVD, PN
Young et al (2003)^52^	LEA	stat. sign.: ethnicity (African American vs White: RR 1.41 [95% CI 1.34‐1.48], Hispanic vs White: 1.28 [1.20‐1.38], Native American vs White: 1.74 [1.39‐2.18], Asian vs White: 0.31 [0.19‐0.50]), nephropathy (3.41 [3.13‐3.71]), diabetic end‐stage renal disease (3.77 [3.57‐3.99])	Age, gender, ethnicity, CVD, hypertension, COPD, service connection, region, stroke, nephropathy, diabetic end‐stage renal disease
Younis et al (2018)^53^	FU	stat. sign.: age (OR 1.027 [95% CI 1.003‐1.051], *P* = .025), duration of diabetes (1.063 [1.027‐1.100], *P* = .001), PN (23.926 [5.41‐105.6], *P* = .001), PVD (0.267 [0.143‐0.532], *P* = .001), HbA1c (6.187 [4.646‐8.239], *P* = .001); not stat. sign.: gender, BMI	Age, gender, duration of diabetes, BMI, HbA1c, PN, PVD
Zhao et al (2016)^54^	FU	stat. sign.: serum cystatin C (OR 4.828 [95% CI 1.711‐13.620], *P* = .003), coronary artery disease (3.566 [1.470‐8.648], *P* = .005), insulin use (2.605 [1.258‐5.394], *P* = .01), difference between supine and sitting transcutaneous oxygen pressure (1.076 [1.032‐1.122], *P* = .001), hypertension (1.021 [1.003‐1.039], *P* = .023); not stat. sign.: age, diastolic blood pressure, haemoglobin, creatinine, calcium, albumin, triglycerides, HDL, proteinuria, microalbuminuria, ABI, transcutaneous oxygen pressure (in sitting position, in supine position)	Age, gender, duration of diabetes, smoking, insulin use, hypertension, coronary artery disease, diastolic blood pressure, haemoglobin, potassium, proteinuria, microalbuminuria, ABI, transcutaneous oxygen pressure (in sitting position, supine position and difference between supine and sitting position)

Abbreviations: ABI, ankle‐brachial index; ALT, alanine transaminase; AST, aspartate transaminase; BMI, body mass index; BP, blood pressure; BUN, blood urea nitrogen; CA, Charcot arthropathy; CI, confidence interval; COPD, chronic obstructive pulmonary disease; CVD, cardiovascular disease; DF, diabetic foot; ECG, electrocardiogram; eGFR, estimated glomerular filtration rate; FG, foot gangrene; FU, foot ulceration; GGT, gamma‐glutamyl transferase; GP, general practitioner; HbA1c, haemoglobin A1c; HDL, high‐density lipoprotein; HR, hazard ratio, LDL, low‐density lipoprotein; LEA, lower extremity amputation; mmHg, millimetres of mercury; OHA, oral hypoglycaemic agent; OR, odds ratio, PlGF, placenta growth factor; PN, peripheral neuropathy; PVD, peripheral vascular disease; RR, risk ratio; stat. sign., statistically significant; T1DM, type 1 diabetes mellitus; T2DM, type 2 diabetes mellitus; VEGF‐A, vascular endothelial growth factor A; VPT, vibration perception threshold; WBC, white blood cell count.

The findings of the single publications were brought together in Table 4 to build an overview of the associations that have been shown for the single risk factors across all included publications. In total, the relationship between 79 different risk factors and the five previously defined outcomes has been studied. Apart from male gender, peripheral neuropathy (PN), retinopathy, nephropathy, poor glycaemic control, insulin use, duration of diabetes, smoking and height, for all of which a positive association with the outcome of interest was shown, the results for the other risk factors showed higher discordances. A total of 41 risk factors were each analysed in one study only.

**TABLE 4:** Risk factor associations oversight

Risk factor	Total number of studies	Studies showing stat. sign. association	Number of studies showing association				Comment
			Any DF	FU	LEA	CA	
Demographic characteristics							
Age	21	11	1^25^	3^24^, ^34^, ^53^	7^41^, ^42^, ^43^, ^45^, ^46^, ^48^, ^50^	1^34^	8 studies showed a positive association,^25^, ^41^, ^42^, ^43^, ^45^, ^46^, ^48^, ^53^ while 3 studies found a negative association^24^, ^34^, ^50^ and 10 studies showed no association with age.^27^, ^28^, ^30^, ^36^, ^37^, ^38^, ^39^, ^40^, ^47^, ^54^
Gender	14	11	1^25^	2^28^, ^39^	9^28^, ^30^, ^38^, ^41^, ^42^, ^43^, ^45^, ^48^, ^50^	0	In 11 studies, a consistently positive association with male gender was shown,^25^, ^28^, ^30^, ^38^, ^39^, ^41^, ^42^, ^43^, ^45^, ^48^, ^50^ while 3 studies showed no association with the outcome of interest.^37^, ^46^, ^53^
Ethnicity/nationality	8	6	1^37^	0	5^30^, ^31^, ^42^, ^50^, ^52^	0	In one study, non‐Saudi nationality was associated with a higher risk for foot complications compared to Saudi nationality.^37^ Other studies found higher risk in American vs European,^31^ Maori vs European,^42^ Malay vs Chinese,^50^ and Africa American, Hispanic or Native American vs White.^52^ Furthermore, 3 studies showed lower risk associated with Asian ethnicity vs White/European^30^, ^42^, ^52^ and Indian vs White/European.^42^ In 5 studies, no association was detected with ethnicity when analysing the following: ethnicity in general,^24^ African American, Hispanic or Mixed vs White,^30^ Pacific vs European,^42^ black and other vs white^44^ and India and other vs Chinese.^50^
Location (urban vs rural)	2	2	0	1^39^	1^45^	0	A positive association was shown in two studies.^39^, ^45^
Living alone (yes vs no)	1	0	0	0	0	0	No association was shown in one study.^39^
Socio‐economic position	4	2	0	0	2^45^, ^48^	0	One study showed a positive association between low income and the outcome^45^; another study showed a positive association with low socio‐economic position, while not detecting an association with low household income.^48^ Two other studies did not find an association.^24^, ^39^
Glycaemic control							
Poor glycaemic control/HbA1c	10	6	1^25^	2^27^, ^53^	3^42^, ^43^, ^44^	0	In 6 studies, a positive association of the outcome with poor glycaemic control/high HbA1c values was found,^25^, ^27^, ^42^, ^43^, ^44^, ^53^ while 4 studies showed no association.^30^, ^37^, ^39^, ^40^
Fasting blood/plasma glucose	4	2	0	0	2^38^, ^46^	0	2 studies showed positive association,^38^, ^46^ while 2 studies showed no association with the outcome of interest.^27^, ^39^
Postprandial blood glucose	1	1	0	1^39^	0	0	A positive association was shown in one study.^39^
Diabetes treatment							
Insulin use	8	5	1^25^	2^39^, ^54^	2^30^, ^35^	0	5 studies showed increased risk for DF complications with insulin use,^25^, ^30^, ^35^, ^39^, ^54^ while 3 studies showed no effect of insulin use^37^, ^40^, ^44^ or no effect of treatment modality in general (insulin vs OHA vs diet).^27^
OHA use	6	1	0	0	1^35^	0	One study showed a negative association and therefore protective effect with metformin use.^35^ Furthermore, no association was detected for use of OHAs,^37^, ^40^, ^44^ clopidogrel,^37^ statins, fibrate/niacin,^30^ glitazones, gliptins and sulphonylureas.^35^ One study showed no effect of treatment modality in general (insulin vs OHA vs diet).^27^
Characteristics of diabetes							
Duration of diabetes	14	8	2^25^, ^37^	2^27^, ^53^	4^30^, ^42^, ^46^, ^48^	0	8 studies showed a positive association with the outcome of interest,^25^, ^27^, ^30^, ^37^, ^42^, ^46^, ^48^, ^53^ while 6 studies showed no association with the duration of diabetes.^24^, ^36^, ^39^, ^40^, ^43^, ^44^
Type of diabetes	5	3	0	0	3^43^, ^46^, ^48^	0	While only one study showed a positive association with T2DM compared to T1DM,^43^ two other studies showed a negative association^46^, ^48^ and two studies found no association.^24^, ^44^
Dyslipidaemia							
Total cholesterol	3	0	0	0	0	0	3 studies consistently showed no association between cholesterol and the outcome of interest.^38^, ^39^, ^44^
HDL‐cholesterol	5	3	0	1^39^	2^30^, ^42^	0	While 2 studies showed a positive association between low values of HDL‐cholesterol and the outcome of interest,^39^, ^42^ one study found a negative association.^30^ In addition, two studies detected no association.^36^, ^54^
LDL‐cholesterol	3	1	0	0	1^30^	0	One study showed a positive association with the outcome,^30^ while two studies detected no association.^36^, ^54^
Triglycerides	6	2	0	1^36^	1^30^	0	While one study showed a positive association^30^ and one study showed a negative association,^36^ 4 other studies reported no association.^38^, ^39^, ^40^, ^54^
Dyslipidaemia	4	2	0	1^47^	1^41^	0	In one study, a positive association was found between dyslipidaemia and the outcome^47^ ^;^ in one study, a negative association was shown with hyperlipidaemia.^41^ Two studies could not detect an association between dyslipidaemia and the outcome of interest.^43^, ^46^
Lifestyle habits							
Smoking	6	3	0	1^39^	2^42^, ^46^	0	While 3 studies showed a positive association with smoking,^39^, ^42^, ^46^ no association was detected in further 3 studies.^30^, ^38^, ^44^
Alcohol	4	2	0	2^27^, ^40^	0	0	While 2 studies showed a positive association with alcohol intake,^27^, ^40^ no association with this potential risk factor was detected in two other studies.^25^, ^38^
Exercise	1	0	0	0	0	0	No association was shown in one study.^27^
Body characteristics							
Hypertension/blood pressure	14	11	0	4^27^, ^34^, ^47^, ^54^	7^30^, ^38^, ^41^, ^42^, ^43^, ^46^	1^34^	While 8 studies found a positive association between hypertension/blood pressure and the outcome of interest,^27^, ^30^, ^34^, ^43^, ^44^, ^46^, ^47^, ^54^ a negative association and therefore a protective effect were shown in two other studies.^38^, ^41^ In one study, a U‐shaped association was detected: both high and low values of systolic blood pressure were associated with higher risk of the outcome.^42^ In 3 studies, no association was found.^25^, ^39^, ^40^
Pulse pressure	1	1	0	1^27^	0	0	A positive association was shown in one study.^27^
BMI/weight	10	4	0	2^39^, ^47^	2^30^, ^43^	0	While two studies showed a positive association,^43^, ^47^ one study found a negative association and therefore a protective effect of higher values of BMI/weight.^39^ In addition, one study showed a negative association of obese vs normal weight, while no association was found for over‐ and underweight vs normal weight.^30^ No association was shown in 6 other studies.^34^, ^38^, ^40^, ^42^, ^44^, ^53^
Height	3	3	0	0	3^30^, ^42^, ^46^	0	A positive association was shown in three studies.^30^, ^42^, ^46^
Diabetic complications, concomitant complications							
Number of diabetic complications	1	1	0	0	1^45^	0	A positive association was shown in one study.^45^
PN/elevated VPT	14	12	1^25^	7^24^, ^27^, ^28^, ^34^, ^40^, ^47^, ^53^	5^28^, ^30^, ^41^, ^43^, ^44^	1^34^	While a consistently positive association was found in 12 studies,^24^, ^25^, ^27^, ^28^, ^30^, ^34^, ^40^, ^41^, ^43^, ^44^, ^47^, ^53^ two studies were not able to find an association.^36^, ^39^
PVD	11	9	1^37^	6^27^, ^28^, ^34^, ^36^, ^39^, ^53^	3^28^, ^41^, ^43^	0	While a positive association with the outcome of interest was found in 7 studies,^27^, ^28^, ^36^, ^37^, ^39^, ^41^, ^43^ one study found a negative association and therefore a protective effect of PVD.^53^ In one study, while a positive association was found between PVD and the outcome FU, no association was shown with the outcome CA.^34^ In two other studies, no association with the respective outcome was shown.^25^, ^54^
CVD	3	2	0	1^27^	1^41^	0	While one study showed a positive association,^27^ one study showed a negative association and therefore a protective effect.^41^ In one study, no association was shown.^25^
CAD	3	1	0	1^54^	0	0	One study showed a positive association with FU,^54^ while two studies detected no association.^25^, ^34^
Myocardial infarction	3	2	0	1^28^	2^28^, ^30^	0	While 2 studies showed a positive association,^28^, ^30^ no association was found in one study.^44^
Heart failure	2	2	0	1^28^	1^41^	0	In one study, a positive association was shown for the outcome LEA.^41^ In another study, a positive association was shown for the outcome FU, but not for the outcome LEA.^28^
Stroke	4	2	0	0	2^30^, ^44^	0	While a positive association was found in two studies,^30^, ^44^ no association was shown in two other studies.^28^, ^41^
Angina/ischaemic heart disease	1	0	0	0	0	0	No association was shown in one study.^28^
History of vascular bypass	1	0	0	0	0	0	No association was shown in one study.^27^
History of angiography	1	0	0	0	0	0	No association was shown in one study.^40^
Arterial fibrillation	1	0	0	0	0	0	No association was shown in one study.^41^
Intermittent claudication	2	2	0	2^27^, ^39^	0	0	A positive association was shown in two studies.^27^, ^39^
Retinopathy	8	8	1^25^	4^27^, ^28^, ^34^, ^39^	4^28^, ^30^, ^41^, ^44^	0	While 7 studies showed a consistently positive association between retinopathy and the outcome,^25^, ^27^, ^28^, ^30^, ^39^, ^41^, ^44^ there was one study that showed a positive association with the outcome FU while showing no association with the outcome CA.^34^
Blindness/impaired vision	1	1	0	0	1^28^	0	In one study, a positive association was found with the outcome LEA, while no association was found with the outcome FU.^28^
Nephropathy	9	6	0	3^27^, ^34^, ^39^	3^30^, ^50^, ^52^	0	While a positive association was shown in 5 studies,^27^, ^30^, ^39^, ^50^, ^52^ no association was found in 3 studies.^25^, ^41^, ^43^ In one further study, while a positive association was shown with the end‐point FU, no association was detected with the outcome CA.^34^
Microalbuminuria	3	1	0	0	1^28^	0	In one study, while a positive association was shown with the outcome LEA, no association was detected with the outcome FU.^28^ Two other studies found no association.^27^, ^54^
Proteinuria	2	0	0	0	0	0	No association was shown in two studies.^28^, ^54^
Cancer	1	0	0	0	0	0	No association was shown in one study.^28^
Mental disorder	2	0	0	0	0	0	No association was shown in two studies.^28^, ^47^
Depression	1	1	0	1^49^	0	0	A positive association was shown in one study.^49^
Social deprivation	1	1	0	1^26^	0	0	A positive association was shown in one study.^26^
Plantar pressure	1	1	0	1^40^	0	0	A positive association was shown in one study.^40^
Flatfoot deformity	1	0	0	0	0	0	No association was shown in one study.^40^
Hallux valgus	1	0	0	0	0	0	No association was shown in one study.^40^
Oxford shoes	1	0	0	0	0	0	No association was shown in one study.^40^
Varicosis	1	0	0	0	0	0	No association was shown in one study.^40^
Dry skin	1	0	0	0	0	0	No association was shown in one study.^40^
Skeletal abnormalities	1	0	0	0	0	0	No association was shown in one study.^40^
Aortic augmentation index	1	0	0	0	0	0	No association was shown in one study.^47^
Charcot joint	1	0	0	0	0	0	No association was shown in one study.^25^
Mediasclerosis	1	1	0	1^40^	0	0	A negative association was shown in one study.^40^
Laboratory parameters							
Haemoglobin	2	1	0	1^39^	0	0	While a positive association was found in one study,^39^ no association was shown in another study.^54^
Serum cystatin C	1	1	0	1^54^	0	0	A positive association was shown in one study.^54^
Bilirubin	3	2	0	2^32^, ^36^	0	0	While, for total bilirubin, one study showed a positive association,^36^ another study showed no association.^39^ In a third study, a positive association was found only for indirect bilirubin, but not for direct and total bilirubin.^32^
C‐reactive protein	1	1	0	1^36^	0	0	A positive association was shown in one study.^36^
Creatinine	3	0	0	0	0	0	No association was shown in three studies.^36^, ^39^, ^54^
Albumin	2	0	0	0	0	0	No association was shown in two studies.^39^, ^54^
Calcium	1	0	0	0	0	0	No association was shown in one study.^54^
Uric acid	1	1	0	1^51^	0	0	A positive association was shown in one study.^51^
WBC	1	0	0	0	0	0	No association was shown in one study.^39^
VEGF‐A	1	1	0	1^33^	0	0	A negative association was shown in one study.^33^
PIGF	1	1	0	1^33^	0	0	A negative association was shown in one study.^33^
Skin autofluorescence	1	1	0	1^36^	0	0	A positive association was shown in one study.^36^
Transcutaneous oxygen pressure	1	1	0	1^54^	0	0	A positive association was shown in one study.^54^
Adherence to preventive measures							
Patient's motivation	1	1	0	1^29^	0	0	While a negative association was shown between the patient's motivation and the outcome FU, no association was found with the outcome LEA.^29^
Patient's effort	1	1	0	1^29^	1^29^	0	A negative association was shown in one study.^29^
Patient's life circumstances	1	1	0	0	1^29^	0	While a negative association was shown between the patient's life circumstances and LEA, no association was found for FU.^29^
Not attending preventive programme	1	1	0	0	1^45^	0	A positive association was shown in one study.^45^
Attending clinic for regular care is not a metabolic disease clinic	1	1	0	0	1^45^	0	A positive association was shown in one study.^45^
Other							
Ownership of hospital for regular care	1	1	0	0	1^45^	0	A positive association was shown in one study.^45^
Low city household income	1	1	0	0	1^45^	0	A positive association was shown in one study.^45^
Year of discharge	1	1	0	0	1^50^	0	A negative association was shown in one study.^50^
Year of amputation	1	1	0	0	1^48^	0	A negative association was shown in one study.^48^

Abbreviations: BMI, body mass index; CA, Charcot arthropathy; CAD, coronary artery disease; CVD, cardiovascular disease; DF, diabetic foot; FU, foot ulceration; HbA1c, haemoglobin A1c; HDL, high‐density lipoprotein; LDL, low‐density lipoprotein; LEA, lower extremity amputation; OHA, oral hypoglycaemic agent; PlGF, placenta growth factor; PN, peripheral neuropathy; PVD, peripheral vascular disease; stat. sign., statistically significant; T1DM, type 1 diabetes mellitus; T2DM, type 2 diabetes mellitus; VEGF‐A, vascular endothelial growth factor; VPT, vibration perception threshold; WBC, white blood cell count.

The assessment of the quality of the included studies using the Newcastle‐Ottawa Quality Assessment Forms for Cohort Studies and Case‐Control Studies yielded results ranging from six to nine out of nine possible stars. Table 5 depicts the risk of bias in the included studies as assessed using the Cochrane handbook guidelines. Although, in a number of cases, some aspects could not be assessed, none of the included studies showed a risk of bias in more than one category.

**TABLE 5:** Risk of various bias in included studies

Publication	Selection bias	Detection bias	Attrition bias	Reporting bias	Other	Comments
Abbott et al (1998)^24^	High	Unclear	Low	Low	Low	Only subjects with PN included in study; not sure if DF conditions present at baseline
Al‐Rubeaan et al (2015)^25^	Unclear	Low	N/A	Low	Low	Only hospital‐based data analysed, which renders generalizability to the general diabetic population unclear; attrition bias not applicable due to cross‐sectional design
Anderson et al (2018)^26^	Low	Low	Low	High	Low	Potential variability in data entry of different GP practices results in potential for under‐reporting of FUs
Baba et al (2014)^27^	Low	Low	Low	High	Low	Limiting the outcome to hospitalizations for FUs may lead to a shift towards patients with more severe courses of FU
Bruun et al (2013)^28^	Low	Low	Low	Unclear	Low	It remains unclear whether the occurrence of FUs between the scheduled visits has been detected
Bruun et al (2014)^29^	Low	Low	Low	Low	Low	
Callaghan et al (2011)^30^	Low	Low	Unclear	Low	Low	No information on proportion of patients lost to follow‐up given
Chaturvedi et al (2001)^31^	Low	Unclear	High	Low	Low	Unclear if DF conditions were present prior to LEA; patient who were lost to follow‐up differed from other subjects (eg were older)
Chen et al (2017)^32^	Unclear	Unclear	N/A	Low	Low	Only hospital‐based data analysed, which renders generalizability to the general diabetic population unclear; unclear if FU present at baseline; attrition bias not applicable due to cross‐sectional design
Chen et al (2018)^33^	Unclear	Unclear	N/A	Low	Low	Only hospital‐based data analysed, which renders generalizability to the general diabetic population unclear; unclear if FU present at baseline; attrition bias not applicable due to cross‐sectional design
Dekker et al (2016)^34^	Unclear	Unclear	Unclear	Low	Low	Various parameters of subjects’ characteristics not stated; therefore, selection bias cannot be judged; unclear if DF conditions were present prior to baseline; no information on proportion of patients lost to follow‐up given
Hippisley‐Cox et al (2016)^35^	Low	Unclear	Low	Low	Low	Unclear if DF conditions were present prior to LEA
Hu et al (2012)^36^	Unclear	Unclear	N/A	Low	Low	Only hospital‐based data analysed, which renders generalizability to the general diabetic population unclear; unclear if FU present at baseline; attrition bias not applicable due to cross‐sectional design
Hu et al (2014)^37^	Unclear	Unclear	N/A	Low	Low	Only hospital‐based data analysed, which renders generalizability to the general diabetic population unclear; unclear if FU present at baseline; attrition bias not applicable due to cross‐sectional design
Humphrey et al (1996)^38^	Low	Unclear	Low	Low	Low	Unclear if DF conditions were present prior to LEA
Jiang et al (2015)^39^	Low	Unclear	Low	Low	Low	Only hospital‐based data analysed, which renders generalizability to the general diabetic population unclear; unclear if DF conditions were present prior to baseline
Kästenbauer et al (2001)^40^	Low	Low	Low	Low	High	Only very small number of subjects (n = 10) developed the outcome of interest
Lai et al (2015)^41^	Low	Unclear	Low	Low	Low	Unclear if DF conditions were present prior to LEA
Robinson et al (2016)^42^	Low	Unclear	Low	Low	Low	Unclear if DF conditions were present prior to LEA
Sarfo‐Kantanka et al (2019)^43^	Unclear	Low	Low	Low	Low	Only hospital‐based data analysed, which renders generalizability to the general diabetic population unclear
Selby et al (1995)^44^	Low	Unclear	Low	Low	Low	Unclear if DF conditions were present prior to LEA
Sheen et al (2018)^45^	Unclear	Unclear	Low	Low	High	Various parameters of subjects’ characteristics not stated; therefore, selection bias cannot be judged; unclear if DF conditions were present prior to LEA
Tseng et al (2006)^46^	Low	Unclear	N/A	High	Low	Unclear if DF conditions were present prior to LEA; attrition bias not applicable due to cross‐sectional design; outcome defined by patients' self‐reported history of surgery
Tuttolomondo et al (2017)^47^	Unclear	Unclear	N/A	Low	Low	Only hospital‐based data analysed, which renders generalizability to the general diabetic population unclear; unclear if FU present at baseline; attrition bias not applicable due to cross‐sectional design
Venermo et al (2013)^48^	Low	Low	Low	Low	Low	
Williams et al (2010)^49^	Low	Low	High	Low	Low	Large number of subjects lost to follow‐up
Yang et al (2011)^50^	Unclear	Unclear	N/A	Low	Low	Various parameters of subjects’ characteristics not stated; therefore, selection bias cannot be judged; unclear if DF conditions were present prior to LEA; attrition bias not applicable due to cross‐sectional design
Ye et al (2014)^51^	High	Unclear	N/A	Unclear	Low	Multivariate analysis only performed in female subpopulation of subjects; only hospital‐based data analysed, which renders generalizability to the general diabetic population unclear; unclear if DF conditions were present prior to baseline; attrition bias not applicable due to cross‐sectional design; unclear if outcome was self‐reported or not
Young et al (2003)^52^	High	Unclear	Low	Low	Low	Subjects in Veterans study do not represent general diabetic population, furthermore various parameters of subject characteristics not stated; unclear if DF conditions were present prior to LEA
Younis et al (2018)^53^	Low	Unclear	N/A	Low	Low	Unclear if DF conditions were present prior to baseline; attrition bias not applicable due to cross‐sectional design
Zhao et al (2016)^54^	Low	Unclear	N/A	Low	Low	Unclear if DF conditions were present prior to baseline; attrition bias not applicable due to cross‐sectional design

Abbreviations: DF, diabetic foot; FU, foot ulceration; GP, general practitioner; LEA, lower extremity amputation; N/A, not assessable; PN, peripheral neuropathy. Colors represent low (green), high (red) and unclear/not assessable (grey) risk of bias.

## 4 DISCUSSION

This systematic review was performed to create a list of the associated risk factors for DF analysed in the literature and to combine the published results. The most frequently assessed variables were age, gender, duration of diabetes, hypertension and PN, followed by peripheral vascular disease (PVD), glycaemic control, BMI or weight and nephropathy. Of the 79 variables that were assessed, the following ones were shown to have a positive association with the outcome of interest in at least three publications (with no publications indicating a negative association): male gender, poor glycaemic control, PN, retinopathy and nephropathy, insulin use, duration of diabetes, smoking and height. Using the Newcastle‐Ottawa Assessment Forms, we confirmed the overall good quality of the studies included in this systematic review, although design problems could have affected the results on specific potential risk factors, as discussed in the following chapters on groups of risk factors.

### 4.1 Gender

One of the risk factors for which the highest consistency was retrieved was male gender. Although the prevalence of diabetes in general and especially the one of DF complications is slightly higher for men compared to women,^55^ the effect has been shown to be even more pronounced in 11 out of 14 studies that analysed male gender as a potential risk factors for DF conditions: all of those studies showed a risk ratio of at least 1.5 for male patients with diabetes compared to female patients with diabetes.^25^, ^28^, ^30^, ^38^, ^39^, ^41^, ^42^, ^43^, ^45^, ^48^, ^50^ In three cross‐sectional studies, no significant association was detected between gender and DF.^37^, ^46^, ^53^

### 4.2 Peripheral neuropathy, retinopathy and nephropathy

A similarly strong association with DF was published for PN and retinopathy as well as for nephropathy. A possible explanation for this result could be due to a common physiological origin: diabetic late complications are classified into macrovascular and microvascular diseases, the latter arising from damage of small blood vessels and leading to retinopathies, nephropathies and neuropathies, a crucial prerequisite for DF conditions.^1^, ^3^, ^56^ For PN, a positive relationship with the respective outcome was detected in twelve out of fourteen studies that analysed this association, with risk ratios ranging from 1.05 to 25.4.^24^, ^25^, ^27^, ^28^, ^30^, ^34^, ^40^, ^41^, ^43^, ^44^, ^47^, ^53^ In only two studies, no association was shown.^36^, ^39^ However, while one of those two studies was a cross‐sectional study that did not detect a relationship between elevated vibration perception threshold, an indication of PN, and FU,^36^ the other one observed the patients for a follow‐up time of only one year in order to assess the development of FU, a time period that might probably be too short to detect long‐term complications in a comprehensive manner.^39^ In eight studies that assessed the potential association of retinopathy with DF, a consistently positive relationship was shown.^25^, ^27^, ^28^, ^30^, ^34^, ^39^, ^41^, ^44^ The only limitation in this agreement is that Dekker et al could show this positive association only when analysing the outcome FU but did not detect an association between retinopathy and the outcome CA.^34^ For nephropathy, a positive relationship was shown in six out of nine studies,^27^, ^30^, ^34^, ^39^, ^50^, ^52^ while the other three did not detect an association.^25^, ^41^, ^43^

### 4.3 Glycaemic control

Although a strong positive relationship with poor glycaemic control would be logical for all late complications of diabetes, discrepancies were shown in the results regarding HbA1c values, fasting or postprandial blood and plasma glucose concentrations: for HbA1c, a positive association was shown in six studies,^25^, ^27^, ^42^, ^43^, ^44^, ^53^ while, in four studies, no association could be detected.^30^, ^37^, ^39^, ^40^ In those that detected a positive association, the risk ratios ranged from values close to one (eg Sarfo et al showed a hazard ratio of 1.11 per one unit (%) increase of HbA1c^43^) to odds ratios larger than six.^53^ In addition, of the four studies that analysed fasting blood glucose, only two showed a positive relationship,^38^, ^46^ while two other studies did not find any association.^27^, ^39^ Postprandial glucose was only assessed as a potential risk factors in one study, in which a positive association with the outcome FU was identified. Notably, the study group that described this association between postprandial glucose and FU could not find any association of HbA1c and fasting blood glucose with FU.^39^ When comparing the study characteristics of the articles that showed varying results concerning the relationship between glycaemic control and DF, there is no notable heterogeneity concerning study design, population sizes or other characteristics that could explain the differences in the results.

### 4.4 Age and duration of disease

With being examined in 21 studies, age was the risk factor for which a potential relationship with DF was analysed the most. However, the results are highly inconsistent: while eight studies showed a positive relationship with the respective outcomes,^25^, ^41^, ^42^, ^43^, ^45^, ^46^, ^48^, ^53^ a negative relationship and therefore a protective effect of patients’ age were shown in three studies.^24^, ^34^, ^50^ In addition to that, ten studies could not detect an association between the patients’ age and the presence of foot complications.^27^, ^28^, ^30^, ^36^, ^37^, ^38^, ^39^, ^40^, ^47^, ^54^ Differences between the study characteristics that could explain these contradictory results could not be retrieved. The eight studies showing a positive relationship analysed different end‐points with one study analysing any DF,^25^ one study analysing FU^53^ and six studies analysing LEA.^41^, ^42^, ^43^, ^45^, ^46^, ^48^ Even the three studies that showed a negative relationship analysed different outcomes: while Abbott et al detected a statistically significant negative relationship with the outcome FU (HR 0.957 for each year of age),^24^ Yang et al analysed the outcome LEA (OR 0.8 associated with age ≥ 65 years)^50^ and Dekker et al detected a protective effect of age with the outcomes FU (OR 0.991 for every year increase) and CA (OR 0.964 for every year increase).^34^ Therefore, although age was stated to be an important risk factor for the development of T2DM itself,^57^ this might not be necessarily the case when analysing foot complications. The crucial factor for the DF might not be the patients’ age per se, but rather the duration living with the disease, a factor that of course correlates with the patients’ age in many cases. This hypothesis is strengthened by the fact that studies, in which the relationship between the duration of diabetes and foot complications was assessed, showed a consistently positive association, even after adjusting for age. This association was reported in eight publications,^25^, ^27^, ^30^, ^37^, ^42^, ^46^, ^48^, ^53^ while six groups could not detect a statistically significant relationship.^24^, ^36^, ^39^, ^40^, ^43^, ^44^ Similar results for the development of DF depending on the duration of diabetes have already been highlighted by Monteiro‐Soares et al^17^

### 4.5 Diabetes treatment

When looking at the studies that analysed diabetes treatment and its potential association with foot complications, the picture on a possible influence of insulin use is rather consistent: five out of nine studies detected a positive relationship between insulin and foot complications,^25^, ^30^, ^35^, ^39^, ^54^ and no study showed a negative association. For the use of oral hypoglycaemic agents (OHA), the picture is less consistent: while, in one study, a protective effect was shown with metformin use,^35^ no association was detected with other OHA in several studies.^30^, ^35^, ^37^, ^40^, ^44^ However, these results have to be interpreted with caution since insulin use is associated with patients showing more severe courses of disease and whose blood glucose levels could not be controlled by lifestyle changes or the use of OHA such as metformin.^58^, ^59^, ^60^ Besides that, it might be hypothesized that patient groups from earlier years have not been treated according to current treatment guidelines and might have received insulin treatment at earlier time points during their course of their disease.

### 4.6 Hypertension and dyslipidaemia

Since physiological anomalies such as hypertension and dyslipidaemia are quite common in T2DM,^18^, ^61^ a positive association of hypertension with late complications such as DF conditions might be hypothesized. For hypertension, the majority of studies, namely eight out of 14 that analysed this association, showed a positive relationship.^27^, ^30^, ^34^, ^43^, ^44^, ^46^, ^47^, ^54^ However, in two studies, a protective effect of high levels of blood pressure was described.^38^, ^41^ While one of those studies was a rather small retrospective cohort study with 375 patients, in which neither the mean duration of diabetes nor the follow‐up time was given,^38^ the other study was a large prospective cohort study analysing more than 45,000 subjects. However, also for the latter study, the patients’ duration of disease and the follow‐up time were not stated, and the validity of the results can therefore not be fully assessed.^41^

Dyslipidaemia is often associated with T2DM: when glucose cannot be metabolized by the cells, fats are mobilized, leading to high levels of fatty acids in the bloodstream.^61^ However, it seems that dyslipidaemia is not associated with DF conditions: of four studies that analysed this potential risk factor, a positive association of dyslipidaemia with FU was only found in one cross‐sectional case‐control study,^47^ while, in another study, a protective effect for LEA was shown with hyperlipidaemia.^41^ Two further studies identified no association with the outcome of interest.^43^, ^46^ In addition, the three studies that analysed the effect of increased cholesterol levels at study entry consistently showed no effect.^38^, ^39^, ^44^ For aberrant levels of HDL‐ and LDL‐cholesterol, the results of the studies are highly inconsistent: while, for low levels of HDL‐cholesterol, two studies showed a positive relationship,^39^, ^42^ one study found a negative one^30^ and two studies found no association.^36^, ^54^ For increased levels of LDL‐cholesterol, one study showed a positive association with the outcome LEA,^30^ but two studies detected no association.^36^, ^54^ For high levels of triglycerides, only one out of six studies identified a positive relationship of triglyceride levels >150 mg/dL and LEA.^30^ In contrast, Hu et al showed a negative relationship and therefore a protective effect of high levels of triglycerides.^36^ Although aberrant levels of lipids and hypertension play an important role in the development of T2DM and late complications such as macrovascular damage that can result in myocardial infarction, PVD or stroke,^3^, ^56^, ^62^ it has to be considered that in some articles, it was not possible to distinguish between the diagnosis of dyslipidaemia and/or hypertension and current blood values which can reach normal levels after proper therapy. Therefore, results on dyslipidaemia and/or hypertension as potential risk factors, especially protective results, must be interpreted with caution.

### 4.7 Obesity, physical activity and height

Although obesity and lack of physical activity are two of the major risk factors for the development of T2DM^18^, ^63^ and the biggest part of T2DM might even be attributed to obesity,^64^ those factors do not seem to play a crucial role in the development of DF complications: out of 10 studies that evaluated the association of BMI or weight,^30^, ^34^, ^38^, ^39^, ^40^, ^42^, ^43^, ^44^, ^47^, ^53^ only two identified a positive relationship with the outcome,^43^, ^47^ while one study showed a negative association.^39^ Another study showed a negative association of obese versus normal weight, while no association was found for over‐ and underweight versus normal weight.^30^ In six studies, no association was shown.^34^, ^38^, ^40^, ^42^, ^44^, ^53^ Exercise was only analysed as a risk factor in one study, in which no association was shown with the outcome FU.^27^ Notably, the analysis of a possible association between height and DF complications led to consistent results over three studies, in all of which a positive association was shown with the outcome LEA.^30^, ^42^, ^46^ This might be due to the fact that a taller body implies larger levels of pressure on the limbs or due to neuropathy depending on the length of nerve fibres with longer fibres being more affected than shorter ones.^65^ While Callaghan et al and Robinson et al found height to be significantly associated with LEA even after adjusting for BMI,^30^, ^42^ Tseng et al did not adjust for BMI.^46^

### 4.8 Peripheral vascular disease and cardiovascular disease

Since T2DM is a metabolic syndrome that increases the risk of heart disease and stroke,^63^, ^66^ and more severe courses of disease are in general associated with more late complications, it might be hypothesized that the presence of any DF disease might correlate with patients’ history of PVD and CVD. Concerning history of PVD and its association with DF conditions, there was high consistency: in seven out of 11 studies, a positive relationship was shown.^27^, ^28^, ^36^, ^37^, ^39^, ^41^, ^43^ However, Younis et al found a negative relationship and therefore a protective effect of history of PVD.^53^ In another study, a retrospective cohort study on more than 22,000 patients, a positive relationship was detected with the outcome FU, but not with the outcome CA.^34^ In two further studies conducted by Al‐Rubeaan et al and Zhao et al, no association was detected.^25^, ^54^ Interestingly, one of those two studies, a cross‐sectional cohort study on 411 subjects conducted by Zhao et al, was the only one of three studies analysing the effect of CVD, that showed a positive relationship for this potential risk factor.^54^ Besides that, a prospective cohort study on more than 45,000 patients in Taiwan showed a protective effect which might be explained by the fact that patients diagnosed with CVD usually receive medical treatment such as drugs against hypertension, antiplatelet therapy or lipid‐lowering therapy, thus preventing peripheral arterial insufficiency.^41^ In addition, no effect between CVD and any DF was again stated by Al‐Rubeaan et al.^25^ From a physiological point of view, the protective effect is not expected, since not only PN, but also the damage of blood vessels, which should be advanced in patients with history of PVD and CVD, enhances DF damage, leading to potential necrosis of tissue and the need for amputation.^1^

## 5 CONCLUSION

An important distinction can be made between amenable and nonamenable risk factors: while nonamenable risk factors such as gender, height or duration of disease cannot be changed by the patient and/or the physician, amenable factors are the ones that can be tackled by patients and their physicians in order to reduce the risk for DF complications. The most important amenable risk factors identified by this most up‐to‐date systematic review are glycaemic control and smoking. Those factors could serve to prevent the development of DF complications and especially the potential for limb amputations, thereby increasing the quality of life of patients with T2DM. Due to the high personal and financial burden associated with DF and the large heterogeneity among included studies, additional longitudinal studies in large patient populations are necessary to identify more modifiable risk factors that can be used in the prediction and prevention of DF complications.

## CONFLICTS OF INTEREST

None declared.

## AUTHOR CONTRIBUTION

SR developed the protocol, conducted the literature search and wrote the first draft of the manuscript. WO was involved in study design, screening of relevant articles, design of result tables and writing the article. ML contributed her clinical expertise to writing the introduction, results and discussion section. All authors have read and approved the final manuscript.

## ETHICAL APPROVAL

The study was conducted in accordance with the Declaration of Helsinki and approved by the Research Committee for Scientific Ethical Questions at UMIT University.

## Data Availability

All relevant data are included in the manuscript.
